# Selective PI3Kδ inhibitor TYM-3-98 suppresses AKT/mTOR/SREBP1-mediated lipogenesis and promotes ferroptosis in KRAS-mutant colorectal cancer

**DOI:** 10.1038/s41419-024-06848-7

**Published:** 2024-07-03

**Authors:** Ya-nan Zheng, Si-yue Lou, Jun Lu, Fan-li Zheng, Yong-mei Tang, En-jun Zhang, Sun-liang Cui, Hua-jun Zhao

**Affiliations:** 1 School of Pharmaceutical Sciences, Zhejiang Chinese Medical University, Hangzhou, China; 2 Academy of Chinese Medical Sciences, Zhejiang Chinese Medical University, Hangzhou, Zhejiang China; 3 Department of Pharmacology, Zhejiang University School of Medicine, Hangzhou, People’s Republic of China; 4 Institute of Drug Discovery and Design, College of Pharmaceutical Sciences, Zhejiang University, Hangzhou, China

**Keywords:** Colon cancer, Pharmacogenetics

## Abstract

Colorectal cancer (CRC) is one of the most common tumors of the digestive system worldwide. KRAS mutations limit the use of anti-EGFR antibodies in combination with chemotherapy for the treatment of CRC. Therefore, novel targeted therapies are needed to overcome the KRAS-induced oncogenesis. Recent evidence suggests that inhibition of PI3K led to ferroptosis, a nonapoptotic cell death closely related to KRAS-mutant cells. Here, we showed that a selective PI3Kδ inhibitor TYM-3–98 can suppress the AKT/mTOR signaling and activate the ferroptosis pathway in KRAS-mutant CRC cells in a concentration-dependent manner. This was evidenced by the lipid peroxidation, iron accumulation, and depletion of GSH. Moreover, the overexpression of the sterol regulatory element-binding protein 1 (SREBP1), a downstream transcription factor regulating lipid metabolism, conferred CRC cells greater resistance to ferroptosis induced by TYM-3–98. In addition, the effect of TYM-3–98 was confirmed in a xenograft mouse model, which demonstrated significant tumor suppression without obvious hepatoxicity or renal toxicity. Taken together, our work demonstrated that the induction of ferroptosis contributed to the PI3Kδ inhibitor-induced cell death via the suppression of AKT/mTOR/SREBP1-mediated lipogenesis, thus displaying a promising therapeutic effect of TYM-3–98 in CRC treatment.

## Introduction

Colorectal cancer (CRC) is the third most common malignancy and the third deadliest cancer worldwide [1]. At present, chemotherapy is one of the most common treatments for CRC, including drugs such as 5-fluorouracil, irinotecan, oxaliplatin, leucovorin, and capecitabine. In addition, treatment using monoclonal antibodies against VEGF and EGFR in combination with chemotherapy has also improved the outcome of CRC [2]. However, these treatment strategies still face challenges ranging from cancer recurrence and drug resistance to overt toxicity [3]. Thus, there is an urgent need for more effective therapies, especially for patients with KRAS or NRAS sequence variations, who lack effective targeted therapies.

PI3K–AKT–mTOR signaling pathway plays an important role in cell growth and survival under physiological and pathological conditions [4], and its overactivation is of great importance during the initiation and development of CRC [5]. In addition, PTEN, a negative regulator of the PI3K pathway, is commonly absent in CRC primary tumors and is associated with an augmented death risk and poor overall survival [6]. Therefore, the PI3K pathway is a promising therapeutic target in the treatment of CRC [7].

Recent studies have reported that pancreatic ductal adenocarcinoma (PDA) patients with KRAS G12R mutations have longer overall survival compared to patients without G12R mutation, but this advantage is offset by concomitant PI3K alterations [8]. The therapeutic vulnerability of PDA patients could be predicted by the profile of the KRAS/PI3K genome, suggesting that targeting PI3K in KRAS mutations may be a novel strategy for the treatment of CRC.

PI3K can be divided into three subtypes based on their structure and substrate specificity: class I, class II, and class III. Among these kinases, only class IA PI3Ks plays a role in human cancer [9]. However, pan-class I inhibitors had strong side effects, including rash and fatigue, hyperglycemia, and potentially limiting dose escalation that may result in sub-optimal PI3K inhibition. Therefore, isoform-specific PI3K inhibitors are developed to improve the prognosis and treatment selection [10]. Class I PI3K consists of a p110 catalytic subunit and a p85 regulatory subunit. The p110 catalytic subunit has four isomers: p110α, p110β, p110δ, and p110γ, encoded by *PIK3CA*, *PIK3CB*, *PIK3CD*, and *PIK3CG* gene, respectively [11]. Though *PIK3CD* is mainly expressed in leukocytes and plays a critical role in some hematological malignancies [12], reports have also shown that its deregulation is associated with solid tumors, including glioma [13, 14], neuroblastoma [15], and breast cancer [16]. In colon cancer tissues and CRC cell lines, PI3Kδ was overexpressed and was an independent predictor for the overall survival of patients with colon cancer, thus suggesting PI3Kδ as a potential therapeutic target for CRC [17].

Ferroptosis is a novel type of iron-dependent programmed cell death, and it is morphologically, biochemically, and genetically different from other types of regulated cell deaths, such as necrosis or apoptosis [18]. In mammalian cells, solute carrier family 7 member 11 (SLC7A11) imports cystine for the synthesis of reduced glutathione (GSH), which is used as a cofactor by glutathione peroxidase-4 (GPX4) to reduce iron accumulation and lipid peroxides to suppress ferroptosis [19]. Studies have shown that ferroptosis inducers that inhibit GPX4 or SLC7A11 can eliminate CRC or enhance the effects of other therapies [20]. Therefore, the study of the occurrence and mechanism of ferroptosis can provide new ideas for the treatment of CRC. The PI3K–AKT–mTOR pathway has recently been shown to be linked to ferroptosis. Inhibition of PI3K, AKT, or mTOR has been shown to sensitize cancer cells to ferroptosis by decreasing sterol regulatory element-binding protein 1 (SREBP1), a central transcription factor regulating lipid metabolism, subsequently inhibiting stearoyl-CoA desaturase-1 (SCD1)-mediated synthesis of monounsaturated fatty acids (MUFA), which induces ferroptosis [21]. Therefore, ferroptosis induction by inhibition of PI3K is a promising approach for the treatment of cancer. However, whether PI3Kδ inhibition can induce ferroptosis in CRC remains to be investigated.

In this study, we found a novel indozole derivative TYM-3–98 that can efficiently inhibit PI3Kδ, which triggeres ferroptosis in CRC via the PI3K–AKT–mTOR-SREBP1 signaling pathway and suppresses the growth of KRAS-mutant CRC both in vitro and in vivo. Our study provides an experimental basis for the use of PI3Kδ inhibitors in the treatment of CRC through the induction of ferroptosis.

## Materials and methods

### Chemicals and reagents

TYM-3–98 was from Dr. Sunliang Cui (College of Pharmaceutical Sciences, Zhejiang University). Idelalisib was purchased from MCE (Cat No. HY-13026). MTT was purchased from Sigma-Aldrich (MO, USA). C11-BODIPY, DFO, and CMC-Na were obtained from Selleck (TX, USA). Antibodies against FTH1 (#4393), GAPDH (#5174), p-mTOR (#5536), mTOR (#2938), p-AKT (#4060), AKT (#4691), p-S6 (#4858s), S6 (#2217s), p-ERK (#9101), and ERK (#9102) were purchased from Cell Signaling Technology (MA, USA). Antibodies against SREBP1 (#sc-13551) was acquired from Santa Cruz Biotechnology (TX, USA) and antibodies against CDO1 (ab232699), SLC7A11 (ab175186), and GPX4 (ab125066) were purchased from Abcam (Cambridge, UK).

### Cell lines and cell culture

Cell lines, including HCT 116, LoVo, and SW620, were purchased from the Cell Bank of the Chinese Academy of Sciences (Shanghai, China), and the cells were recently authenticated by STR profiling. HCT 116, LoVo, SW620 cells were cultured in DMEM medium supplemented with 10% FBS. All cell cultures were supplemented with 1% penicillin/streptomycin and incubated at 37 °C in a humidified atmosphere of 5% CO_2_.

### Cell viability assay

Cell viability was assessed by MTT assay. Briefly, cells were seeded at densities of 3 × 10^3^ cells per well, and incubated with different compounds at indicated concentrations for 48 or 72 h. 20 µL of MTT were added to each well, and 4 h later, DMSO was added to each well to dissolve the formazan crystals. Absorbance was measured using a microplate reader (Bio-Tek, CA, USA) at 570 nm. GraphPad Prism 5.0 (GraphPad Software) was used for curve fitting and IC_50_ calculation.

### Western blotting

Cells or snap-frozen tumor samples were lysed in RIPA buffer at 4 °C for 30 min. After centrifugation at 16,000×*g* at 4 °C for 15 min, the supernatant was collected, and the protein concentration was detected with the BCA protein detection kit (Beyotime, Shanghai, China). Western blotting was performed as described previously [22].

### Proteomic analysis

HCT 116 cells were inoculated into a six-well plate with 1.5 × 10^5^ cells per well, and treated with TYM-3–98 at different concentrations for 48 h. Cells were then collected and washed twice with PBS. 4% SDS containing protease inhibitor was added to the cell pellet. The cell lysate was then collected and the protein concentration was determined by the BCA protein detection kit. The samples were then sent to the Institute of Basic Medicine and Cancer, The Cancer Hospital of the University of Chinese Academy of Sciences (Zhejiang Cancer Hospital) for further proteomic analysis.

### In vivo tumor xenograft study

Male BALB/c nude mice aged 4–6 weeks were purchased from Shanghai Nanfang Model Biotechnology Co. LTD (Shanghai, China). The experiment was conducted in strict accordance with institutional ethical guidelines on animal care. In total, 5 × 10^6^ HCT 116 cells were injected subcutaneously into the right flank of each mouse. When the tumor volume reached 50 mm^3^, the mice were randomly divided into five groups (*n* = 6): control, TYM-3–98 (5 mg/kg, 10 mg/kg, 15 mg/kg), and Idelalisib (15 mg/kg). sTYM-3–98 and Idelalisib were administered intragastrically once per day. The mice’s weight and tumor volume were measured every other day. After 21 days, the mice were sacrificed, and tumors, livers, and kidneys were removed for further studies. Tumor volume was calculated as: 0.5 × length (mm) × width^2^ (mm^2^).

### Measurement of lipid peroxidation

In total, 1 × 10^5^ cells per well were seeded into a six-well plate. After treatment with various concentrations of TYM-3–98 or Idelalisib for the indicated period of time, the cells were collected, washed twice with PBS, and then dyed with C11-BODIPY for 30 min at 37 °C in the dark. After being resuspended with PBS, the cells were filtered into single-cell suspension and analyzed on a Guava Easy Cytometer.

### Intracellular GSH and total iron assay

Cells were seeded in six-well plates with 1 × 10^6^ cells per well. After TYM-3-98 treatment for 24 h, the GSH levels were measured using a GSH detection kit (No. S0053, Beyotime, Shanghai), while total iron levels were measured using an iron detection kit (E1042, Applygen, Beijing).

### Measurement of ALT/AST

Serum levels of alanine aminotransferase (ALT, #C009–2–1, Jiancheng, Nanjing) and aspartate aminotransferase (AST, #C010–2–1, Jiancheng, Nanjing) were measured according to the manufacturer’s instructions for each kit.

### Lentiviral-mediated SREBP1 overexpression

The plasmid containing the insert SREBP1 was obtained from Guannan Biotechnology Co. LTD (Hangzhou, China). Lentiviruses were produced by the co-transfection of the lentiviral vector with packaging plasmids into 293FT cells using Lipofectamine 2000 (Thermo Fisher Scientific), and transfection of target cells is done as described previously [23].

### Transient transfection

To knockdown PI3Kδ, different siRNAs against human PI3Kδ (siRNA-1: 5′‐GCGCCAAGATGTGCCAATT-3′; siRNA-2: 5′‐GCAACGAGTGCTGTGCAA‐3′; siRNA-3: 5′‐GCCAAGAUGUGCCAAUUTT‐3′) were designed, and nonspecific siRNA (NC siRNA) was used as a negative control. The siRNA duplexes and negative control (NC) (scrambled siRNA) were synthesized and purified by OBIO Tech (Shanghai, China). For siRNA transfection, 2 × 10^5^ CRC cells per well were seeded in 24-well plates in a serum-free medium. After 24 h, PI3Kδ siRNA or NC siRNA was transfected into cells using Lipofectamine 2000 transfection reagents (Thermo Fisher Scientific) following the manufacturer’s instructions.

### shRNA plasmid transfection

The shRNAs targeting the *SREBF1* gene were purchased from OBIO Tech (Shanghai, China). The sequences of the shRNAs are as follows: sh*SREBF1*–1 5′-CAGGITATGCCGAGTTTACATGACCT-3′; sh*SREBF1*–2 5′-GAGAGTACATCGTCAATATTCCGCG-3′; sh*SREBF1*–3 5′-CCAAAGCTTGAGCACTCAACTGCT-3′. Transfection of cells was performed using Lipofectamine 2000 reagent (Thermo Fisher Scientific) according to the manufacturer’s instructions.

### Hematoxylin and eosin (HE) staining and immunohistochemistry (IHC) analysis

Xenograft tumor tissue, liver, and kidney samples were collected, fixed with formalin overnight, and embedded in paraffin. IHC staining was performed on 5-μm thick sections using antibodies against PI3Kδ (1:500), SREBP1 (1:500), and p-AKT (1:500). For HE staining, the slides were stained with hematoxylin–eosin.

### qRT-PCR

Total RNA was extracted by Trizol (Invitrogen, Carlsbad, CA) after treatment with TYM-3–98. The mRNA was reverse transcribed into cDNA, and qRT-PCR was performed as previously described [22]. The primers used for qRT-PCR in this study are listed in Supplementary Table 1.

### Statistical analysis

All data are expressed as mean ± standard deviation of at least three independent experiments. Statistical significance was analyzed using the Student’s *t* test. The criterion of statistical significance was *P < 0.05, **P < 0.01, ***P < 0.001.

## Results

### TYM-3–98 suppressed the proliferation of CRC cells in vitro via the PI3K–AKT-mTOR signaling pathway

**Fig. 1:**
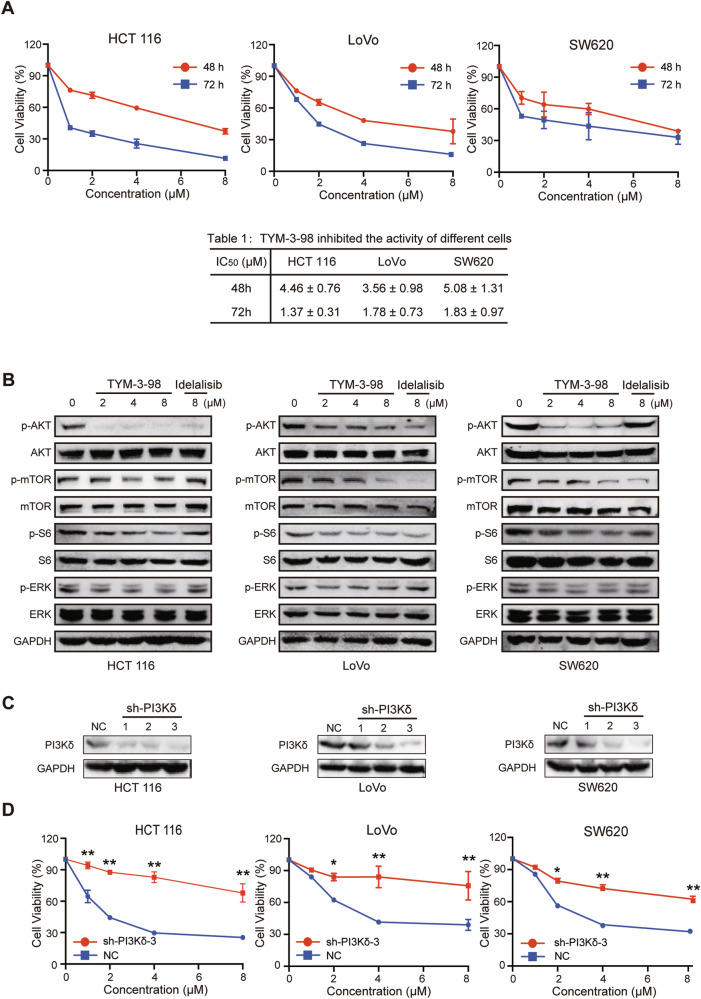
TYM-3-98 suppressed the proliferation of CRC cell lines via the PI3K–AKT–mTOR signaling pathway. **A** HCT 116, LoVo, and SW620 cells were treated with TYM-3-98 for 48 h or 72 h. Cell viability was measured by MTT assay as detailed in “Materials and methods”. IC_50_ values are presented as means ± SD of three independent experiments. **B** Western blotting analysis for PI3K pathway-related proteins in CRC cell lines after treatment with indicated concentrations of TYM-3-98 or Idelalisib. **C** The protein levels of PI3Kδ were detected in CRC cells transfected with siNC or siPIK3CD. **D** CRC cells transfected with siPIK3CD were treated with TYM-3-98 for 48 h and the cell viability was measured by MTT assay. Data are representative of three independent experiments. *P < 0.05, **P < 0.01.

Given the potential importance of PI3Kδ overactivation in CRC [17], and based on the favorable PI3Kδ isoform selectivity of TYM-3–98 (structure is shown in Supplementary Fig. S1A) [24], it was further evaluated in a series of CRC cell lines. As shown in Fig. 1A, in KRAS-mutant HCT 116, LoVo, and SW620 cell lines, TYM-3–98 inhibited the proliferation of all three cell lines in a concentration- and time-dependent manner, with IC_50_ value of 1.37 μM, 1.78 μM, and 1.83 μM (Table 1), respectively, after the 72 h treatment.

**Table 1:** TYM-3-98 inhibited the activity of different cells.

IC_50_(μM)	HCT 116	LoVo	SW620
48 h	4.46 ± 0.76	3.56 ± 0.98	5.08 ± 1.31
72 h	1.37 ± 0.31	1.78 ± 0.73	1.83 ± 0.97

To verify whether the inhibitory effect of TYM-3–98 on the proliferation of CRC cells was due to the inhibition of the PI3K–AKT–mTOR signaling pathway, we evaluated the phosphorylation level of related components within the pathway. As depicted in Fig. 1B, TYM-3–98 concentration-dependently reduced the phosphorylation of AKT (Ser473), mTOR, and S6, which is consistent with the positive control Idelalisib, illustrating that the activity of PI3K–AKT–mTOR pathway in CRC cells is blocked by TYM-3–98. As the inhibition of the PI3K–AKT–mTOR signaling network may result in feedback hyperactivation of MAPK, which promotes cell proliferation and limits the application of PI3K inhibitors [25], we further analyzed the phosphorylation of ERK after PI3K inhibition by TYM-3–98. The result showed that compared to Idelalisib, TYM-3–98 more effectively suppressed the phosphorylation of ERK, thus suggesting a greater capacity to prevent feedback activation of MAPK and a robust anticancer effect in the clinic.

The expression of PIK3CD in CRC cells was knocked down by siRNA to explore whether TYM-3–98-induced cell death is mediated by inhibition of PI3Kδ (Fig. 1C). Results showed that the knockdown of PIK3CD significantly reversed the inhibitory effect of TYM-3–98 on CRC cell viability (Fig. 1D), suggesting that TYM-3–98, to some extent, inhibits CRC cell viability by inhibiting PI3Kδ.

### TYM-3–98-induced ferroptosis in CRC cells

**Fig. 2:**
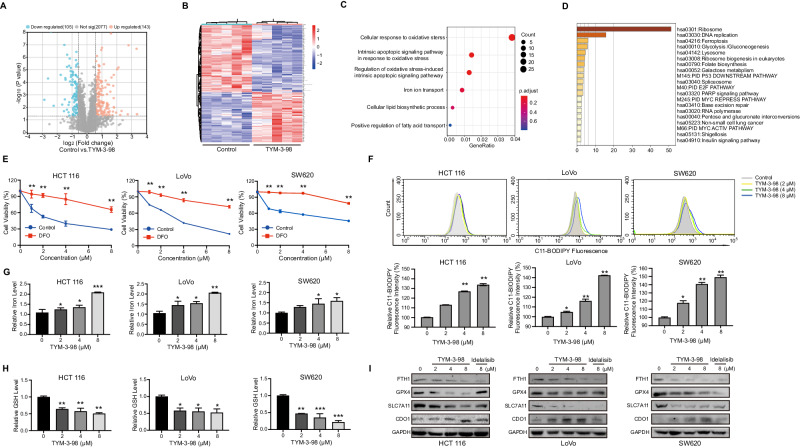
TYM-3-98-induced ferroptosis in CRC cells. **A** Volcano plot depicting the downregulated and upregulated proteins in the TYM-3-98-treated group vs. that of the control group. **B** Differentially expressed proteins in control and TYM-3-98-treated group. **C** KEGG analysis of differentially expressed proteins altered by TYM-3-98. **D** Pathway analysis of the differentially expressed proteins using the Metascape web-based platform. **E** After pre-treatment with or without DFO for 3 h, HCT 116, LoVo, and SW620 cells were exposed to TYM-3-98 for another 48 h. Cell viability was detected by MTT analysis. **F** After treatment with TYM-3-98 for 12 h, the lipid peroxidation levels were evaluated by a C11-BODIPY 581/591 fluorescent probe. **G** After treatment with TYM-3-98 for 24 h, the total iron levels were detected by an iron detection kit. **H** After being treated with TYM-3-98 for 24 h, GSH levels were detected by the GSH kit. **I** Western blotting analysis for ferroptosis pathway-related proteins in CRC cell lines after treatment with TYM-3-98 or Idelalisib with indicated concentrations for 24 h. Data are representative of three independent experiments. *P < 0.05, **P < 0.01, ***P < 0.001.

To further understand the molecular mechanism of the anticancer effect of TYM-3–98, we first measured the ratio of apoptotic cells after TYM-3–98 treatment. To our surprise, unlike previous reports where inhibition of PI3K resulted in the induction of apoptosis in leukemia [26, 27], TYM-3–98 did not significantly increase the level of apoptosis in HCT 116, LoVo, and SW620 cells (Supplementary Fig. S1B). To clarify the mechanism of TYM-3–98-induced cell death, HCT 116 cells treated with TYM-3–98 were sent for proteomics analysis. A total of 248 differentially expressed proteins were detected by comparing the control group and the TYM-3–98-treated group (*P* value < 0.01, fold change >1.5). A heatmap and volcano plot showed that 105 proteins were downregulated and 143 proteins were upregulated in the treated group (Fig. 2A, B). Based on these differentially expressed proteins, we performed bioinformatics analysis to identify the key pathways altered by TYM-3–98. Metascape and KEGG analysis found that the ferroptosis pathway, cellular oxidative stress, and iron ion transport were strongly activated after the administration of TYM-3–98 (Fig. 2C, D). These results suggest that the ferroptosis process may be involved in the proliferation inhibition of TYM-3–98 on CRC.

Ferroptosis is considered as a programmed oxidative cell death, accompanied by lipid peroxidation and iron accumulation, resulting in the production of reactive oxygen species [28]. To verify the proteomic results, we first administered the iron chelator DFO to determine whether ferroptosis has a role in TYM-3–98-induced cell death. As shown in Fig. 2E, the inhibition of ferroptosis by DFO significantly rescued the inhibition of viability after CRC cells treated by TYM-3–98. Further evaluation showed that TYM-3–98 induced a rapid concentration-dependent accumulation of lipid peroxides (Fig. 2F), long with increased intracellular iron and reduced antioxidant GSH within 24 h (Fig. 2G, H). GPX4 is one of the most important antioxidant enzymes and an essential regulator of ferroptotic cell death. Studies have shown that the activation of GPX4 and SLC7A11 can suppress ferroptosis [19, 20, 29]. In agreement with these reports, TYM-3–98 decreased the expression levels of GPX4, SLC7A11, and ferritin heavy chain (FTH1), while increasing cysteine dioxygenase 1 (CDO1) (Fig. 2I). Together, these data suggest that TYM-3–98-induced ferroptosis inhibits CRC cell proliferation in vitro.

Taken together, these results showed that TYM-3–98 could induce ferroptosis in HCT 116, LoVo, and SW620 cells by inhibiting PI3Kδ, thus exerting an anti-CRC property.

### TYM-3–98 inhibited tumor growth in mouse xenograft tumor models

**Fig. 3:**
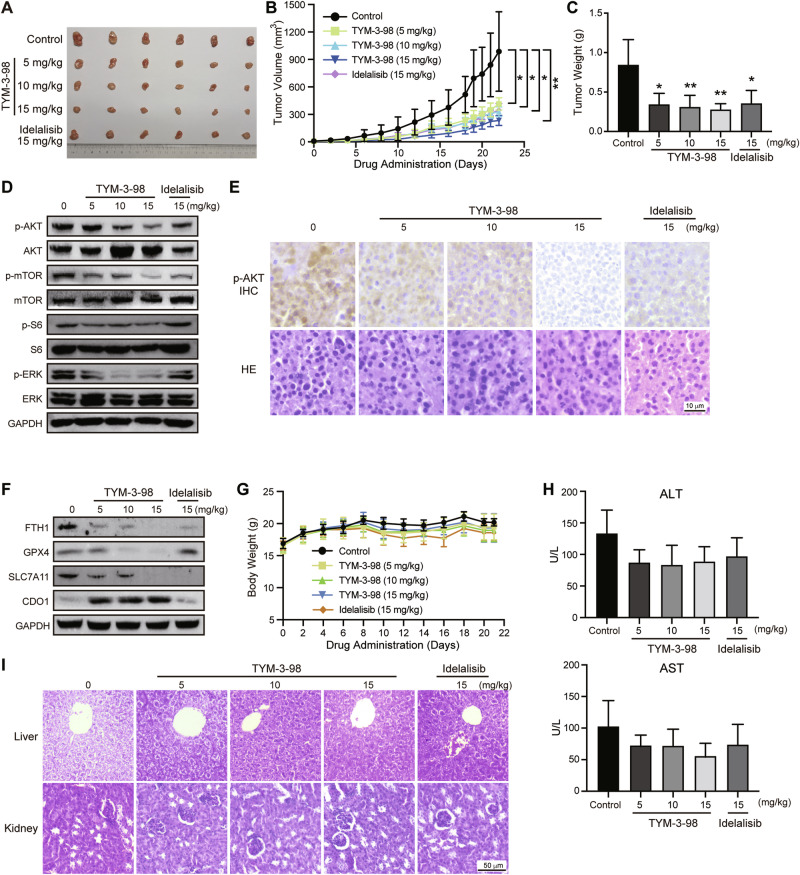
In vivo antitumor activity of TYM-3-98 in HCT 116 xenograft mouse model. **A** Image of HCT 116 tumors from nude mice 21 days after initiation of treatment (*n* = 6). **B** The volume of tumors from sacrificed mice (*n* = 6). **C** Weight of tumors from sacrificed mice (*n* = 6). **D** Western blotting analysis of PI3K pathway-related proteins in tumor tissues (*n* = 3). **E** Representative HE and IHC images of p-AKT, from sections of xenografted tumors (*n* = 3). **F** Western blotting analysis of ferroptosis pathway-related proteins in tumor tissues (*n* = 3). **G** The average body weight changes of tumor-bearing nude mice during the 21-day study (*n* = 6). **H** Alanine aminotransferase (ALT) and aspartate transaminase (AST) levels in serum were determined by ALT/AST kits (*n* = 3). **I** Representative HE staining of liver, and kidney sections in TYM-3-98 and Idelalisib-treated groups. *n* = 3. *P < 0.05, **P < 0.01.

To further investigate the anticancer potential of TYM-3–98 on CRC tumor growth in vivo, nude mice were xenografted with HCT 116 cells. Six days after tumor inoculation, the mice were divided into 5 groups and treated daily with vehicle, TYM-3–98 (5, 10, 15 mg/kg), or Idelalisib (15 mg/kg). Compared with the control and Idelalisib group, TYM-3–98 significantly decelerated tumor growth in a dose-dependent manner (Fig. 3A–C). Similar to what we observed in vitro, TYM-3–98 inhibited PI3K–AKT and MAPK signaling pathways, while activating ferroptosis in xenografted tumors (Fig. 3D–F). In addition, TYM-3–98 produced no observable toxic effects as judged by the weight of the mice (Fig. 3G). Levels of serum ALT, and AST were not significantly different among groups (Fig. 3H). Morphological analysis of both liver and kidney sections showed structurally intact cells with clear nuclei and uniform cytoplasmic staining, with no pathological changes such as inflammatory cell infiltration in the liver, renal cortex, or interstitium, indicating that the treatment was well tolerated (Fig. 3I). Collectively, these results demonstrate that the inhibition of PI3Kδ by TYM-3–98 leds to ferroptosis in vivo, with no significant effect on the hepatic or renal function of the mice.

### SREBP1 mediates TYM-3–98-induced ferroptosis in CRC cells

**Fig. 4:**
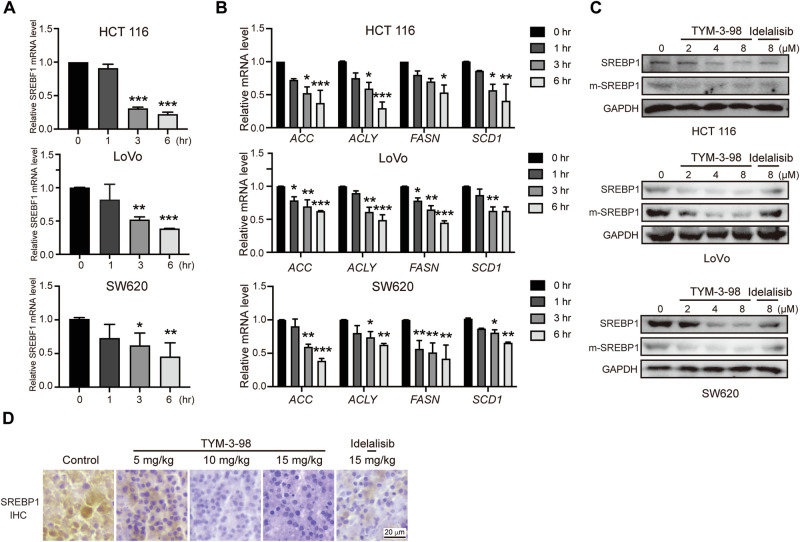
TYM-3-98 inhibited the expression of SREBP1. **A** mRNA level of *SREBF1* was detected by RT-PCR after treatment with TYM-3-98 (8 μM) for indicated times. **B** The mRNA levels of the target genes of SREBP1 were measured by RT-PCR. **C** The expression of total and mature SREBP1 after treatment with TYM-3-98 for 24 h was analyzed by western blotting. **D** Representative IHC staining of SREBP1 from sections of xenografted tumors. Data are representative of three independent experiments. *P < 0.05, **P < 0.01, ***P < 0.001.

Ferroptosis requires intracellular phospholipid peroxidation, and to investigate whether TYM-3–98 regulates ferroptosis by modulating cellular lipid metabolism, we examined SREBP1, a key transcription factor that controls lipogenesis and lipid uptake, which has recently been identified as a downstream target of mTOR [21, 30]. Administration of TYM-3–98 decreased the mRNA expression of *SREBF1* and multiple downstream lipid synthesis-related genes, including *ACC, ACLY, FASN*, and *SCD* (Fig. 4A, B). Consistently, TYM-3–98 decreased the protein levels of both total SREBP1 and its mature form (mSREBP1) both in vitro and in vivo (Fig. 4C, D).

**Fig. 5:**
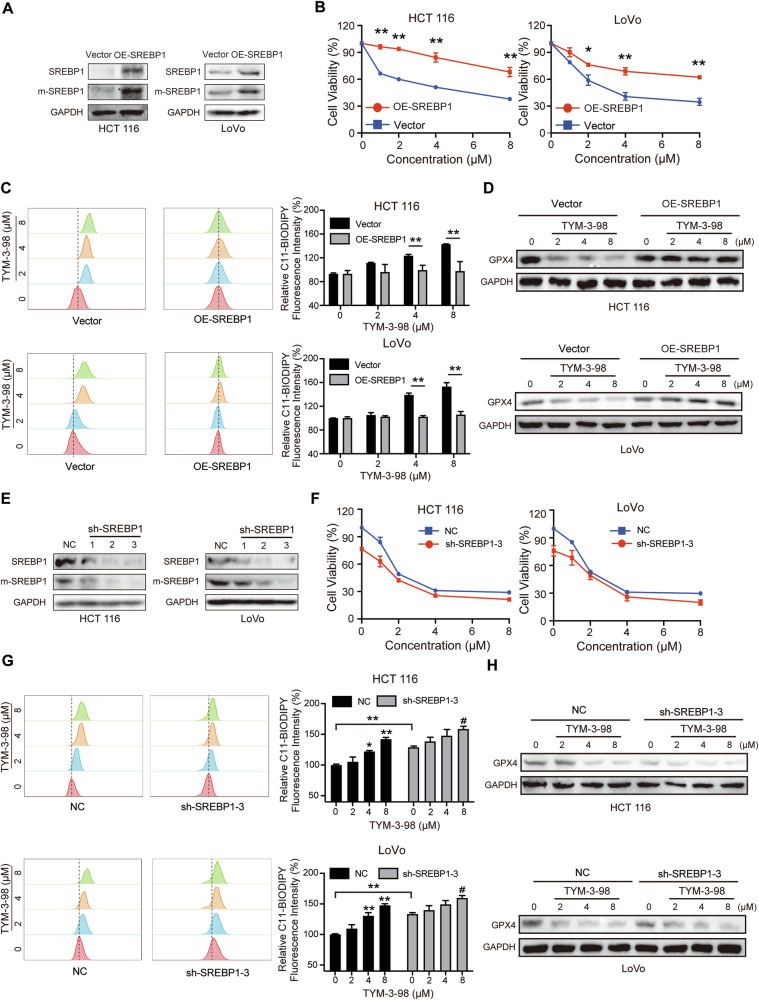
SREBP1 protected CRC cells from TYM-3-98-induced ferroptosis. **A** The level of SREBP1 in vector and SREBP1-overexpressed CRC cells, monitored by western blotting. **B** After overexpression of SREBP1, cells were treated with TYM-3-98 for 24 h, and viability was evaluated by MTT assay. **C** After overexpression of SREBP1 and TYM-3-98 treatment for 12 h, HCT 116, and LoVo cells were stained with C11-BODIPY to measure lipid peroxidation. **D** After overexpression of SREBP1, cells were treated with TYM-3-98 for 24 h and the protein levels of GPX4 were detected by western blotting. **E** The protein levels of SREBP1 and mSREBP1 were detected in CRC cells transfected with shNC or shSREBF1. **F** CRC cells transfected with shSREBF1 were treated with TYM-3-98 for 48 h and the cell viability was measured by MTT. **G** After knocking down SREBP1 and TYM-3-98 treatment for 12 h, HCT 116 and LoVo cells were stained with C11-BODIPY to measure lipid peroxidation. **H** HCT 116 and LoVo cells transfected with shSREBF1 were treated with TYM-3-98 for 24 h and the protein levels of GPX4 were detected by western blotting. *P < 0.05, **P < 0.01, ^#^*P* < 0.05. * vs NC, ^#^ vs sh-SREBP1-3.

To investigate whether SPEBP1 plays a key role in modulating ferroptosis sensitivity, we overexpressed SREBP1 (Fig. 5A) and found that overexpression of SREBP1 restored cell viability against TYM-3–98 treatment (Fig. 5B). Besides that, after overexpression of SREBP1, lipid ROS levels affected by TYM-3–98 were partially restored, as well as changes in ferroptosis-related proteins (Fig. 5C, D). However, the knockdown of SREBP1 in CRC cells induced ferroptosis, and the treatment of TYM-3–98 further inhibited CRC cell viability (Fig. 5E, F). In addition, TYM-3-98 affected the levels of lipid ROS and ferroptosis-related proteins in cells that had been knocked down for SREBP1 (Fig. 5G, H).

The above evidence suggests that TYM-3-98 promotes ferroptosis by suppressing SREBP1 activity in CRC cells. Collectively, these experiments demonstrate that the inactivation of PI3K–AKT-mTOR signaling induces ferroptosis via SREBP1/SCD1-mediated lipogenesis, and that TYM-3-98 is a promising therapeutic agent for the treatment of KRAS-mutated CRC cells.

## Discussion

In this study, we found that TYM-3-98 is a highly selective PI3Kδ inhibitor, showing potent activity against CRC both in vitro and in vivo. TYM-3-98 induces ferroptotic cell death in KRAS-mutant CRC cells via inhibition of the oncogenic PI3K–AKT pathway, while the overexpression of SREBP1 results in resistance of CRC cells to TYM-3-98-induced ferroptosis.

Due to the high frequency of PI3K pathway mutations and dysregulations, the PI3K/AKT/mTOR pathway has been a popular target for antitumor drug research. However, the development of PI3K inhibitors has been limited by modest monotherapeutic efficacy and significant adverse effects. One possible reason for the limited efficacy is the multiple enzyme isoforms that are so closely related [31]. Therefore, isoform-specific PI3Kδ inhibitors have been developed in order to achieve better effects compared to pan or dual PI3K inhibitors because of the toxicity profile, especially for the treatment of B-cell malignancy [32]. Here, we report that the selective PI3Kδ inhibitor TYM-3-98 displays a superior anti-CRC efficacy than the first-generation PI3Kδ inhibitor Idelalisib without showing obvious renal or hepatoxicity in vivo (Fig. 3). Further experiments are needed to clarify the safety profile of TYM-3-98, for example, evaluating the side effects commonly associated with δ isoform such as gastrointestinal toxicity, transaminitis and myelosuppression [7].

Since activation or dysregulation of the class 1 A PI3K signaling pathway is a major contributor to resistance against a variety of anticancer agents, including chemotherapy, radiotherapy, hormone therapy, and targeted agents [33] Compared with the use of PI3K inhibitors as monotherapy, combining them with other anticancer therapies may be a more effective strategy in improving current standard-of-care and clinical outcomes. For example, it was found that combinations of cetuximab and PI3K, AKT, or mTOR inhibitors can profoundly control tumor growth in metastatic CRC regardless of driver genotypes [34, 35]. The combination of PI3K inhibitor and mTOR inhibitor plays a synergistic role in anti-CRC cells by enhancing the inhibition of 4EBP [36]. In addition, the dual inhibition of MAPK and PI3K pathways can also effectively block the progression of CRC cells with KRAS mutations [37]. Therefore, future study may either use TYM-3-98 as monotherapy or in combination with other antitumor agents to optimize the antitumor effect. However, the challenge remains to identify the most rational combinations of those agents with acceptable safety and tolerability.

Ferroptosis, as an iron-dependent form of nonapoptotic cell death, plays an important role in the targeted treatment of CRC [20, 29]. Recently, PI3K–AKT signaling has been reported to be closely related to ferroptosis, and a variety of compounds can either inhibit or induce ferroptosis by regulating this signaling pathway. Du et al. used GEO and TCGA databases to establish prognostic prediction models and explore FRGs-based treatment strategies for CRC patients, and found that the PI3K–AKT signaling pathway, YAK-STAT signaling pathway, Ras signaling pathway and MAPK signaling pathway were closely related to the ferroptosis pathway [38]. KLF2 inhibits the progression of CRC by inducing ferroptosis through the PI3K–AKT signaling pathway [39]. In this study, we found that TYM-3-98 suppresses PI3Kδ and exerts an inhibitory effect on CRC by inducing ferroptosis, which is confirmed by depleted GSH, elevated iron ion levels, and accumulated lipid peroxidation (Fig. 2).

A recent study reported that the inhibition of PI3K–AKT-mTOR signaling sensitizes cancer cells to ferroptosis induction via ablation of SREBP1/SCD1-mediated production of MUFA, which inhibits ferroptosis [21, 40]. This is in parallel with our finding that TYM-3-98 can inhibit CRC by inducing ferroptosis through suppression of SREBP1 and its downstream targets, and that overexpression of SREBP1 attenuated the efficacy of TYM-3-98 (Fig. 5). Nevertheless, further studies are warranted to elucidate whether other activation or inhibition mechanisms of ferroptosis, such as the supply of polyunsaturated fatty acids and derivatives by activating the ACSL4-LPCAT3 pathway, lipophagy or glutaminolysis, may contribute to the ferroptosis induced by TYM-3-98.

In conclusion, TYM-3-98 is a novel selective PI3Kδ inhibitor and a ferroptosis inducer with promising activity against CRC both in vitro and in vivo, making it a potential targeting agent for CRC.

### Supplementary information

Table S1

Original Data

supplementary information file

## Data Availability

The datasets used and/or analyzed during the current study are available from the corresponding author on reasonable request.
