# Supplementary material for: Selective PI3Kδ inhibitor TYM-3-98 suppresses AKT/mTOR/SREBP1-mediated lipogenesis and promotes ferroptosis in KRAS-mutant colorectal cancer
Source: Cell Death Dis. 2024 Jul 3;15(7):474. doi: 10.1038/s41419-024-06848-7 (PMC11220027; doi:10.1038/s41419-024-06848-7)
Supplement: Supplementary file 1 — Table S1 [file 41419_2024_6848_MOESM1_ESM.docx]

**Table S1** : Synthesized primers for qRT-PCR

| Gene | Forward | Reverse |
| --- | --- | --- |
| SCD1 | 5’-TACCGCTGGCACATCAACTT-3’ | 5’-AGTGAACTTCATCAGCGGGG-3’ |
| ACC | 5’-GATGTGGATGATGGGCTACA-3’ | 5’-TGAGGCCTTGATCATTACTGG-3’ |
| ACLY  FASN | 5’-CTCCGGATTTTGCGGGGT-3’  5’-GTTCACGGACATGGAGCAC-3’ | 5’-AGGAGTTCTTTGCCCGTCTG-3’  5’-GTGGCTCTTGATGATCAGGTC-3’ |
| SREBF1 | 5’-GCTGCTGACCGACATCGAA-3’ | 5’-GGGTGGGTCAAATAGGCCAG-3’ |
| GAPDH | 5’-ACAACTTTGGTATCGTGGAAGG -3’ | 5’-GCCATCACGCCACAGTTTC -3’ |
