# Supplementary material for: Selective PI3Kδ inhibitor TYM-3-98 suppresses AKT/mTOR/SREBP1-mediated lipogenesis and promotes ferroptosis in KRAS-mutant colorectal cancer
Source: Cell Death Dis. 2024 Jul 3;15(7):474. doi: 10.1038/s41419-024-06848-7 (PMC11220027; doi:10.1038/s41419-024-06848-7)
Supplement: Supplementary file 2 — Original Data [file 41419_2024_6848_MOESM2_ESM.pdf]

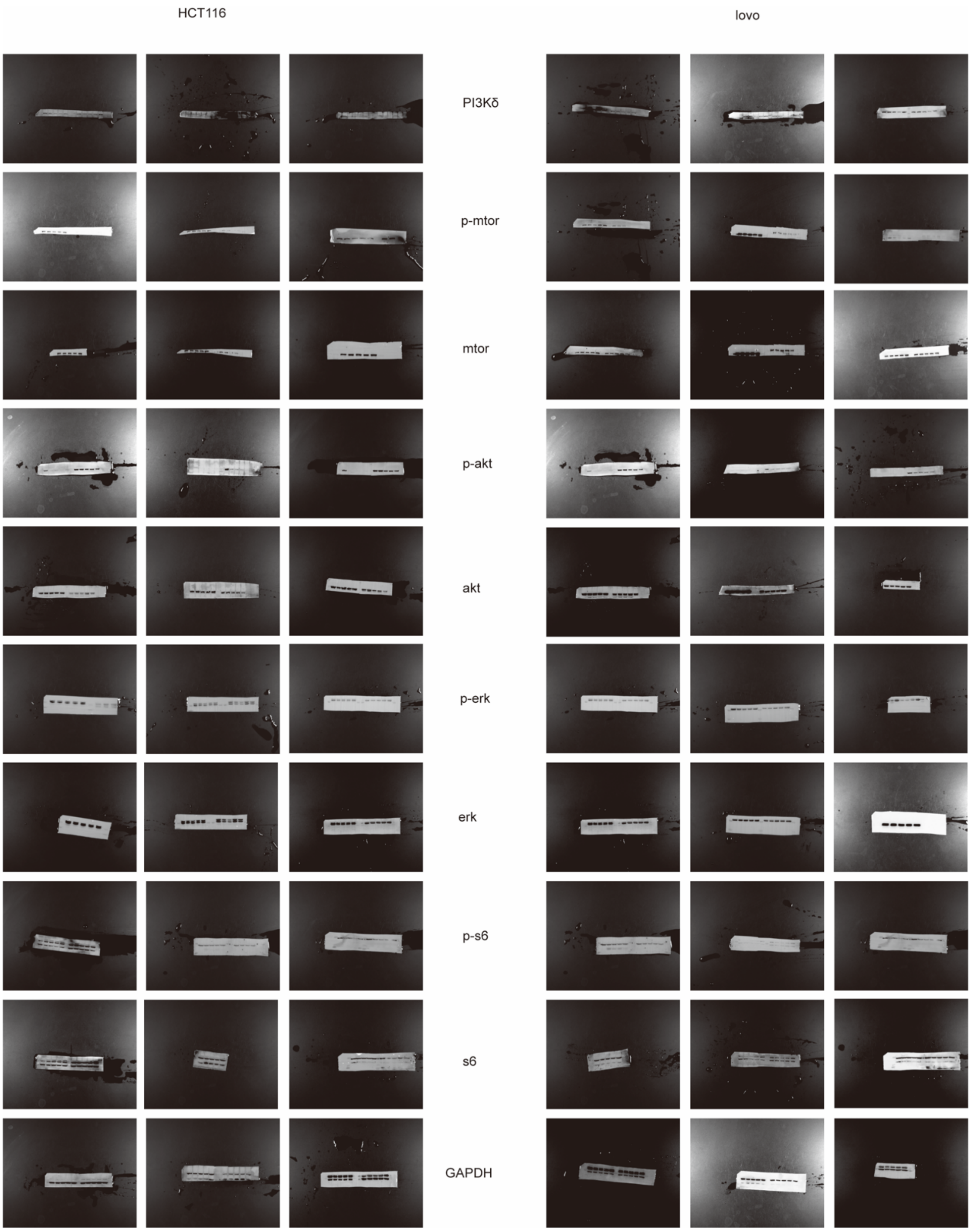
HCT116
lovo
PI3Kδ
p-mtor
mtor
p-akt
akt
p-erk
erk
p-s6
s6
GAPDH

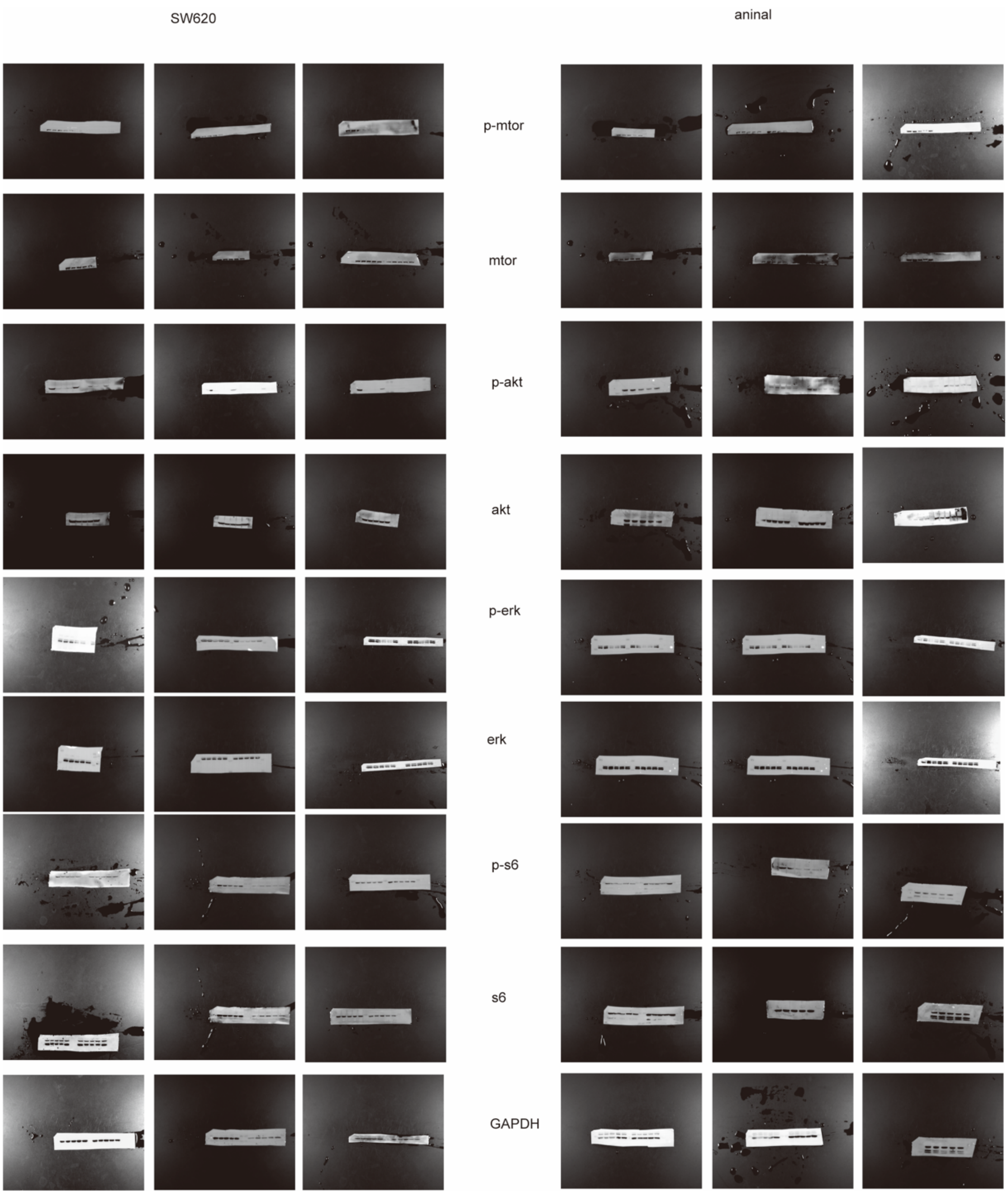
SW620
aninal
p-mtor
mtor
p-akt
akt
p-erk
erk
p-s6
s6
GAPDH

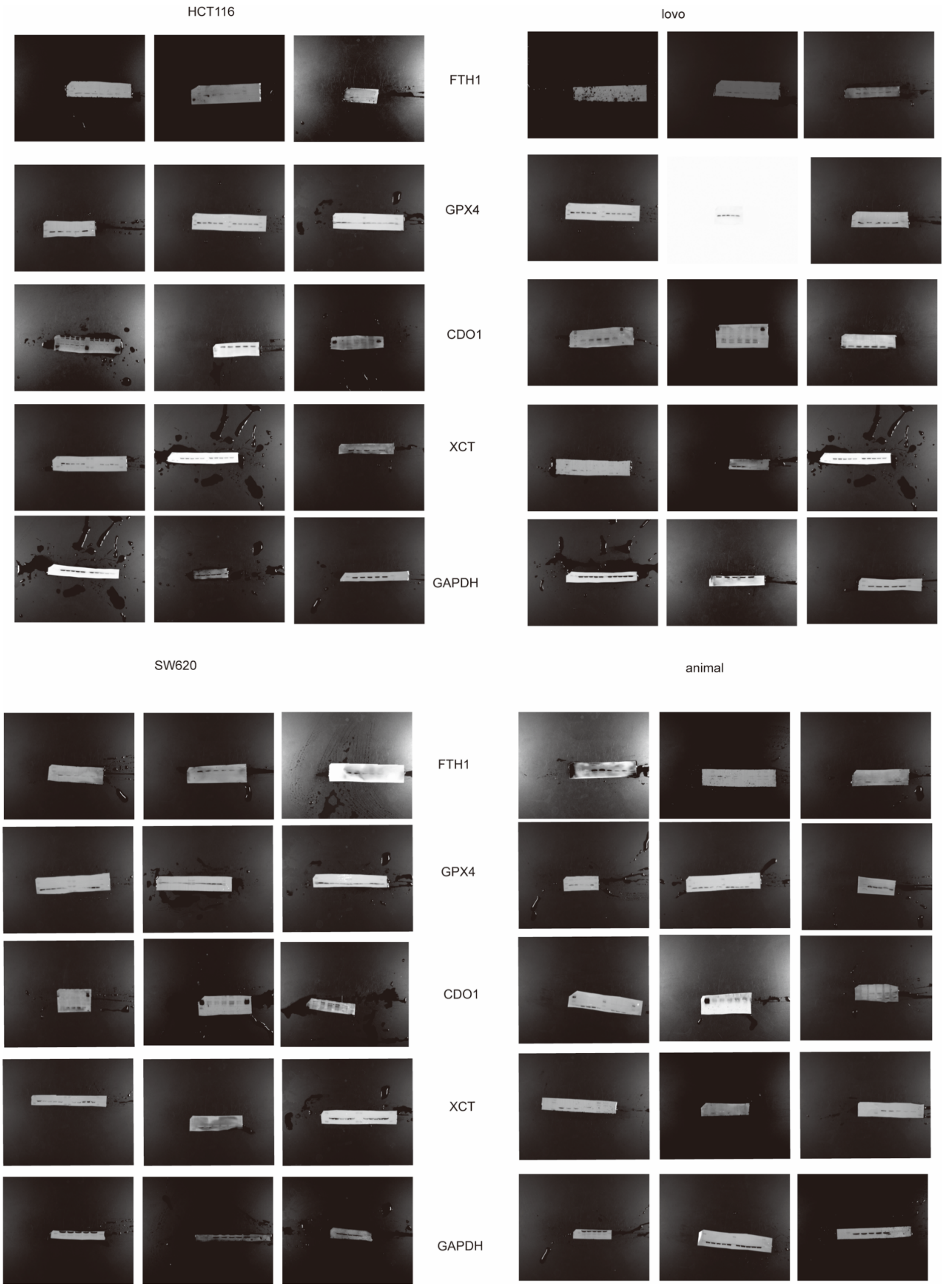
HCT116
lovo
FTH1
GPX4
CDO1
XCT
GAPDH
SW620
animal
FTH1
GPX4
CDO1
XCT
GAPDH

HCT116

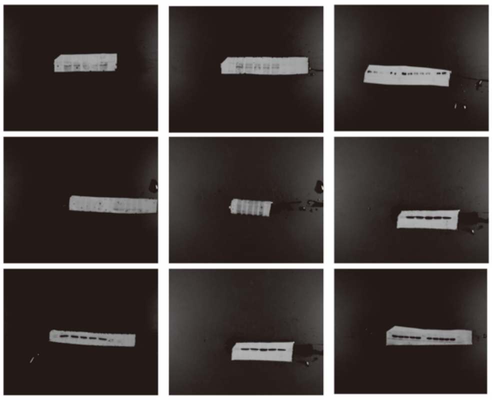

SREBP1

mSREBP1

GAPDH

LoVo

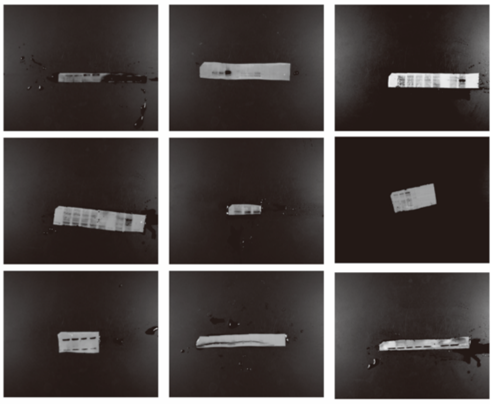

SW620

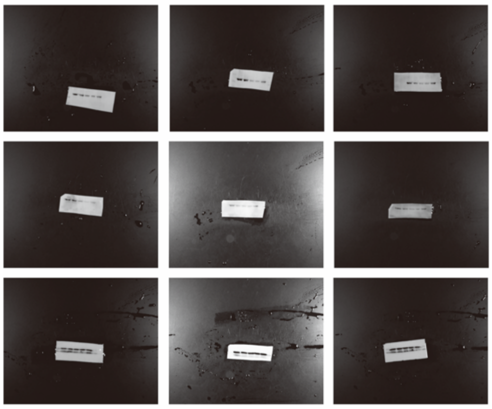

SREBP1

mSREBP1

GAPDH

HCT116 over-expression

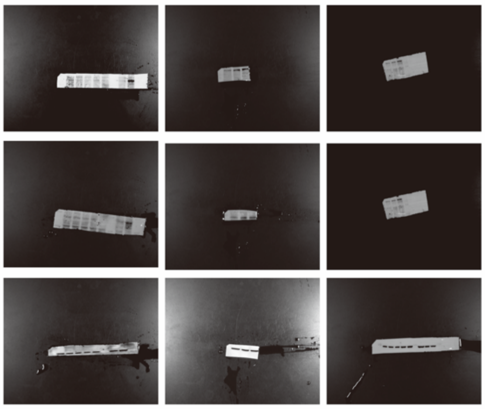

LoVo over-expression

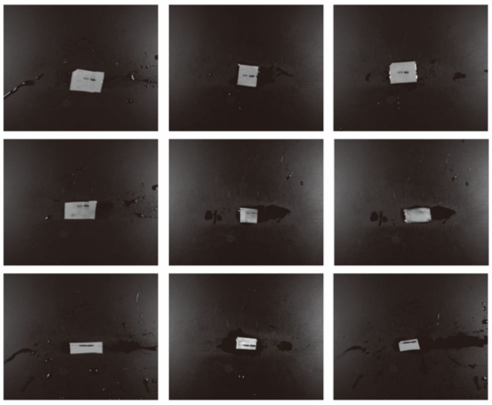

SREBP1

mSREBP1

GAPDH

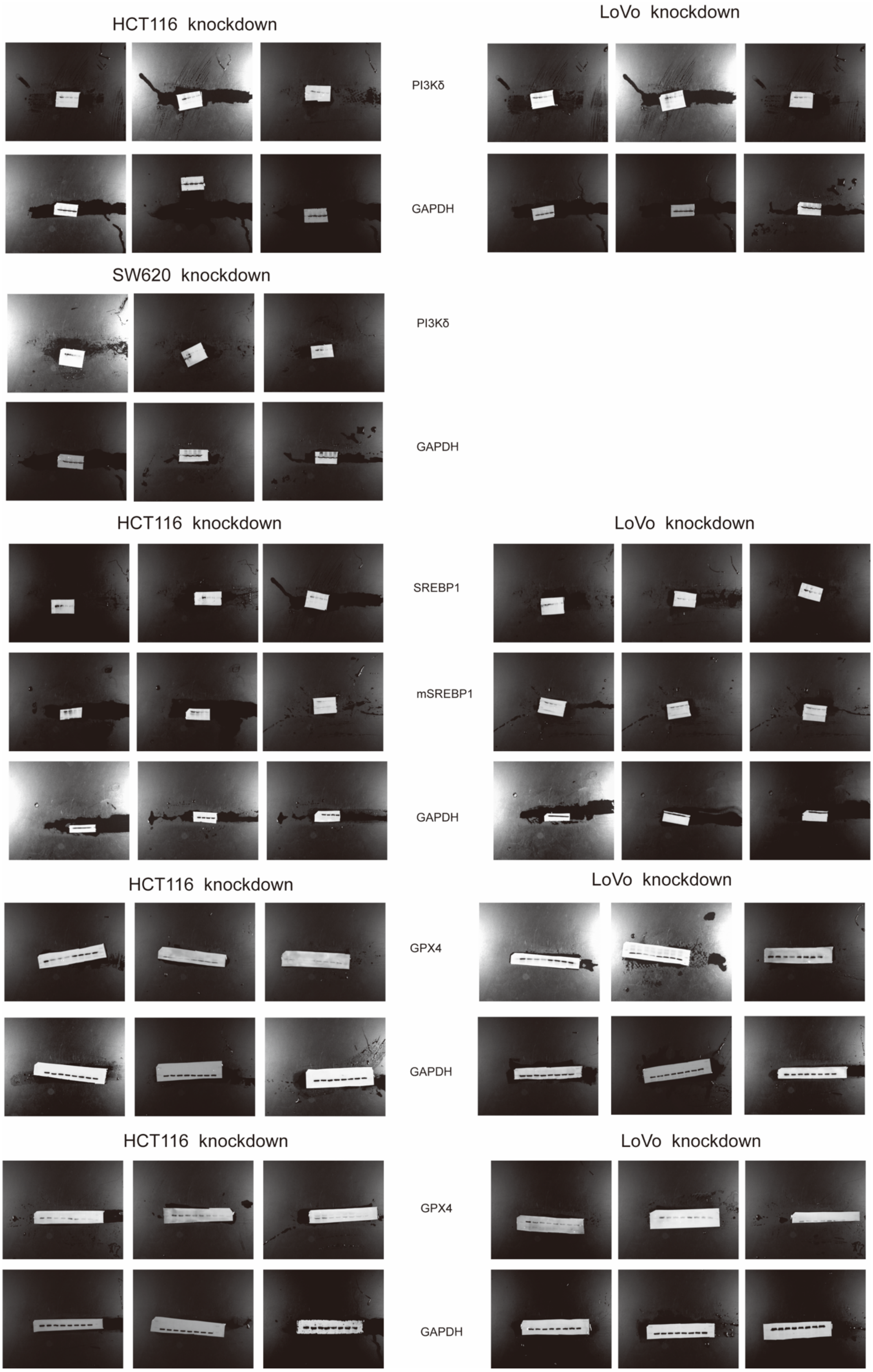
HCT116 knockdown
LoVo knockdown
PI3Kδ
GAPDH
SW620 knockdown
PI3Kδ
GAPDH
HCT116 knockdown
LoVo knockdown
SREBP1
mSREBP1
GAPDH
HCT116 knockdown
LoVo knockdown
GPX4
GAPDH
HCT116 knockdown
LoVo knockdown
GPX4
GAPDH
