# Supplementary material for: Selective PI3Kδ inhibitor TYM-3-98 suppresses AKT/mTOR/SREBP1-mediated lipogenesis and promotes ferroptosis in KRAS-mutant colorectal cancer
Source: Cell Death Dis. 2024 Jul 3;15(7):474. doi: 10.1038/s41419-024-06848-7 (PMC11220027; doi:10.1038/s41419-024-06848-7)
Supplement: Supplementary file 3 — supplementary information file [file 41419_2024_6848_MOESM3_ESM.pdf]

# Supplementary Data

## Supplementary Materials

Detection of cell apoptosis

Apoptosis analysis was performed by Annexin V-FTIC Apoptosis Detection Kit (BD Pharmingen). CRC cells were seeded in six-well plates at 1 × $10^6$ cells per well, and then incubated for 48 h. Cells were collected and resuspended, stained with Annexin V-FITC (5 μL) and PI (5 μL) in the dark for 15 min, and then detected by flow cytometry using Guava Easy Cytometer (Merck, Germany).

## Supplementary Figure

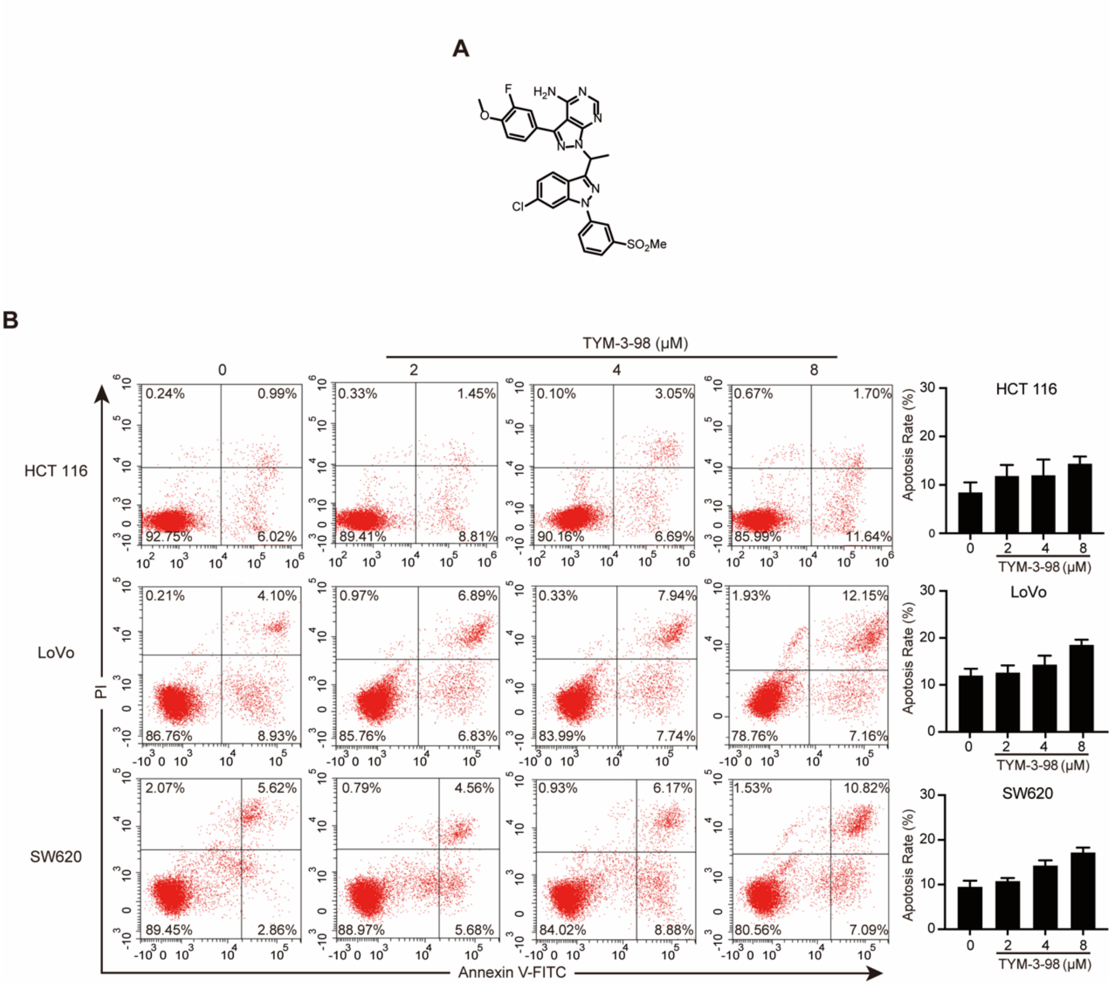


**Figure S1.** (A) Chemical structure of TYM-3-98. (B) Apoptosis rate in TYM-3-98-treated CRC cells was evaluated by Annexin V-FITC/PI staining and flow cytometry analysis.

**Table S1** : Synthesized primers for qRT-PCR

| Gene | Forward | Reverse |
|---|---|---|
| SCD1 | 5'-TACCGCTGGCACATCAACTT-3' | 5'-AGTGAACTTCATCAGCGGGG-3' |
| ACC | 5'-GATGTGGATGATGGGCTACA-3' | 5'-TGAGGCCTTGATCATTACTGG-3' |
| ACLY | 5'-CTCCGGATTTTGCGGGGT-3' | 5'-AGGAGTTCTTTGCCCGTCTG-3' |
| FASN | 5'-GTTCACGGACATGGAGCAC-3' | 5'-GTGGCTCTTGATGATCAGGTC-3' |
| SREBF1 | 5'-GCTGCTGACCGACATCGAA-3' | 5'-GGGTGGGTCAAATAGGCCAG-3' |
| GAPDH | 5'-ACAACTTTGGTATCGTGGAAGG -3' | 5'-GCCATCACGCCACAGTTTC -3' |

# 动物实验伦理审查合格证明

Certificate of Ethical and Welfare Review of Animal Experiments

| 批准编号 Approval No. | 20220613-14 | 终审编号 Review No. | IACUC-202208-01 |
|---|---|---|---|

经过实验动物管理与伦理委员会审核，该项目动物实验过程符合动物保护、动物福利和伦理原则，符合国家实验动物福利伦理的相关规定。

According to the audit of the laboratory animal management and ethics committee, the animal experiment process of this project conforms to the principles of the animal protection, the animal welfare and the ethics as well as the related stipulation on national experimental animal welfare ethics.

| | | | | | |
|---|---|---|---|---|---|
| 实验名称 Protocol Title | 选择性PI3Kδ抑制剂TYM-3-98抑制AKT/mTOR/ srebp1介导的脂肪生成并促进kras突变型结直肠癌中的铁死亡<br>Selective PI3Kδ inhibitor TYM-3-98 suppresses AKT/mTOR/SREBP1-mediated lipogenesis and promotes ferroptosis in KRAS-mutant colorectal | | | | |
| 申请人姓名 Applicant | 郑亚楠<br>Zheng ya nan | 职称/学位 Title/Degree | 硕士<br>master | 邮箱 Email | 499400343@qq.com |
| 课题负责人 Principal Investigator | 赵华军<br>Zhao hua jun | 职称/学位 Title/Degree | 教授<br>Professor | 邮箱 Email | zhj@zcmu.edu.cn |
| 院系（部门） Department | 药学院<br>pharmacy department | | | | |
| 实验时间 Experimental period | 2022-6-28 - 2022-8-1 | 实验动物使用许可证 Number of Animal Use Permit | SYXK（浙）2021-0012 | | |
| 动物实验研究中心主治兽医 LARC Attending Veterinarian | [signature] | | | 日期 Date | 2022.8.8 |
| 动物实验研究中心IACUC主席 LARC IACUC Chairman | 吕建敏 | | | 日期 Date | 2022.8.8 |

浙江中医药大学实验动物管理与伦理委员会
Animal Ethical and Welfare Committee of ZCMU

日期(Date)：2022.8.8

浙江中医药大学 实验动物管理与伦理委员会

# HCT116 STR

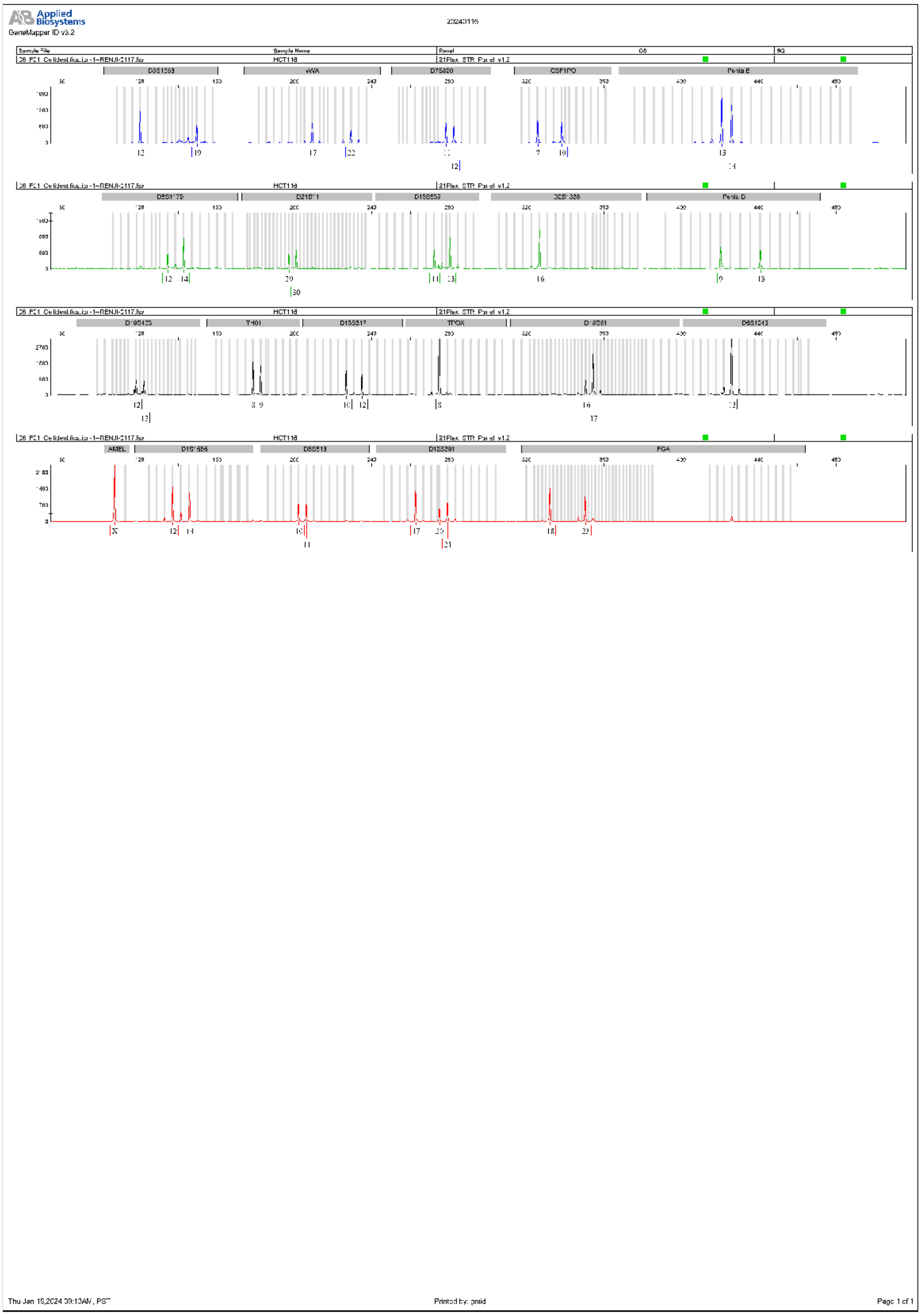
Applied Biosystems
GeneMapper ID v3.2
Sample File
Sample Name
Panel
HCT116
AMEL
FGA
TPOX

| EV | Cell No. | Cell name | Locus names | | | | | | | | |
|---|---|---|---|---|---|---|---|---|---|---|---|
| | | | D5S818 | D13S317 | D7S820 | D16S539 | VWA | TH01 | AM | TPOX | CSF1PO |
| | ***Query (Your Cell)*** | | 10,11 | 10,12 | 11,12 | 11,13 | 17,22 | 8,9 | X,X | 8,8 | 7,10 |
| 0.94(34/36) | HPACC | HCT-116 | ['10', '11'] | ['10', '12'] | ['11', '12'] | ['11', '13'] | ['17', '22'] | ['8', '9'] | ['X', 'Y'] | ['8', '8'] | ['7', '10'] |
| 1.0(36/36) | CVCL_VU38 | HCT 116-Luc2 [ATCC] | ['10', '11'] | ['10', '12'] | ['11', '12'] | ['11', '13'] | ['17', '22'] | ['8', '9'] | ['X', 'X'] | ['8', '8'] | ['7', '10'] |
| 1.0(36/36) | CVCL_XC98 | HCT116-Cas9-566 | ['10', '11'] | ['10', '12'] | ['11', '12'] | ['11', '13'] | ['17', '22'] | ['8', '9'] | ['X', 'X'] | ['8', '8'] | ['7', '10'] |
| 1.0(36/36) | CVCL_XC99 | HCT116-Cas9-567 | ['10', '11'] | ['10', '12'] | ['11', '12'] | ['11', '13'] | ['17', '22'] | ['8', '9'] | ['X', 'X'] | ['8', '8'] | ['7', '10'] |
| 1.0(36/36) | CVCL_XD00 | HCT116-Cas9-568 | ['10', '11'] | ['10', '12'] | ['11', '12'] | ['11', '13'] | ['17', '22'] | ['8', '9'] | ['X', 'X'] | ['8', '8'] | ['7', '10'] |
| 1.0(36/36) | CVCL_XD01 | HCT116-Cas9-TSC22KO-569 | ['10', '11'] | ['10', '12'] | ['11', '12'] | ['11', '13'] | ['17', '22'] | ['8', '9'] | ['X', 'X'] | ['8', '8'] | ['7', '10'] |
| 1.0(36/36) | CVCL_XE63 | HCT116-EGFP | ['10', '11'] | ['10', '12'] | ['11', '12'] | ['11', '13'] | ['17', '22'] | ['8', '9'] | ['X', 'X'] | ['8', '8'] | ['7', '10'] |
| 0.94(34/36) | KCLB-Korea-10247 | HCT-116 | ['10', '11'] | ['10', '12'] | ['11', '12'] | ['11', '13'] | ['17', '22'] | ['8', '9'] | ['X', 'Y'] | ['8', '8'] | ['7', '10'] |
| 0.94(34/36) | CVCL_HG26 | HCT 116 EP300 WT | ['10', '11'] | ['10', '13'] | ['11', '12'] | ['11', '13'] | ['17', '22'] | ['8', '9'] | ['X', 'X'] | ['8', '8'] | ['7', '10'] |
| 0.92(33/36) | CVCL_J254 | HCT-116 clone#2-Luc | ['10', '11'] | ['10', '12'] | ['11', '12'] | ['11', '13', '14'] | ['17', '18', '22', '23'] | ['8', '9'] | ['X', 'X'] | ['8', '8'] | ['7', '10'] |
| 0.89(32/36) | CCL-247 | HCT-116 | ['10', '11'] | ['10', '12'] | ['11', '12'] | ['11', '13'] | ['17', '22'] | ['8', '9'] | ['X', 'Y'] | ['8', '9'] | ['7', '10'] |
| 0.89(32/36) | CRL-2780 | ATRFLOX-[Mutatect] | ['10', '12'] | ['10', '12'] | ['11', '12'] | ['11', '13'] | ['17', '22'] | ['8', '9'] | ['X', 'X'] | ['8', '8'] | ['7', '9'] |
| 0.89(32/36) | CVCL_2299 | ATRFLOX | ['10', '12'] | ['10', '12'] | ['11', '12'] | ['11', '13'] | ['17', '22'] | ['8', '9'] | ['X', 'X'] | ['8', '8'] | ['7', '9'] |
| 0.89(32/36) | CVCL_HG30 | HCT 116 EP300(-) rescued | ['10', '11'] | ['10', '13'] | ['11', '12'] | ['11', '13'] | ['18', '22'] | ['8', '9'] | ['X', 'X'] | ['8', '8'] | ['7', '10'] |
| 0.89(32/36) | CVCL_0291 | HCT 116 | ['10', '11'] | ['10', '12'] | ['11', '12'] | ['11', '13'] | ['17', '22'] | ['8', '9'] | ['X ', 'X'] | ['8 ', '8'] | ['7', '10'] |
| 0.89(32/36) | CVCL_0291 | HCT 116 | ['10', '11'] | ['10', '12'] | ['11', '12'] | ['11', '13'] | ['17', '22'] | ['8', '9'] | ['X ', 'X'] | ['8', '9'] | ['7', '10'] |
| 0.89(32/36) | CVCL_0291 | HCT 116 | ['10', '11'] | ['10', '12'] | ['11', '12'] | ['11', '13'] | ['17', '22'] | ['8', '9'] | ['X', 'Y'] | ['8 ', '8'] | ['7', '10'] |
| 0.89(32/36) | CVCL_0291 | HCT 116 | ['10', '11'] | ['10', '12'] | ['11', '12'] | ['11', '13'] | ['17', '22'] | ['8', '9'] | ['X', 'Y'] | ['8', '9'] | ['7', '10'] |
| 0.86(31/36) | CVCL_0291 | HCT 116 | ['10', '11'] | ['10', '12'] | ['11', '12'] | ['11', '13'] | ['17', '22'] | ['8', '9', '10'] | ['X ', 'X'] | ['8 ', '8'] | ['7', '10'] |
| 0.86(31/36) | CVCL_0291 | HCT 116 | ['10', '11'] | ['10', '12'] | ['11', '12'] | ['11', '13'] | ['17', '22'] | ['8', '9', '10'] | ['X ', 'X'] | ['8', '9'] | ['7', '10'] |
| 0.86(31/36) | CVCL_0291 | HCT 116 | ['10', '11'] | ['10', '12'] | ['11', '12'] | ['11', '13'] | ['17', '22'] | ['8', '9', '10'] | ['X', 'Y'] | ['8 ', '8'] | ['7', '10'] |
| 0.86(31/36) | CVCL_0291 | HCT 116 | ['10', '11'] | ['10', '12'] | ['11', '12'] | ['11', '13'] | ['17', '22'] | ['8', '9', '10'] | ['X', 'Y'] | ['8', '9'] | ['7', '10'] |
| 0.85(31/36) | CVCL_RU11 | PPT2 | ['10', '11'] | ['10', '11', '13'] | ['11', '12'] | ['11', '13', '14'] | ['17', '18', '21', '22'] | ['8', '9'] | ['X', 'X'] | ['8', '8'] | ['7', '10'] |
| 0.84(30/36) | CVCL_0291 | HCT 116 | ['10', '11'] | ['10', '12'] | ['11', '12'] | ['11', '12', '13', '14'] | ['17', '22'] | ['8', '9'] | ['X ', 'X'] | ['8 ', '8'] | ['7', '10'] |
| 0.84(30/36) | CVCL_0291 | HCT 116 | ['10', '11'] | ['10', '12'] | ['11', '12'] | ['11', '12', '13', '14'] | ['17', '22'] | ['8', '9'] | ['X ', 'X'] | ['8', '9'] | ['7', '10'] |
| 0.84(30/36) | CVCL_0291 | HCT 116 | ['10', '11'] | ['10', '12'] | ['11', '12'] | ['11', '12', '13', '14'] | ['17', '22'] | ['8', '9'] | ['X', 'Y'] | ['8 ', '8'] | ['7', '10'] |
| 0.84(30/36) | CVCL_0291 | HCT 116 | ['10', '11'] | ['10', '12'] | ['11', '12'] | ['11', '12', '13', '14'] | ['17', '22'] | ['8', '9'] | ['X', 'Y'] | ['8', '9'] | ['7', '10'] |
| 0.84(30/36) | CVCL_0291 | HCT 116 | ['10', '11'] | ['10', '12'] | ['11', '12'] | ['11', '13'] | ['17', '21', '22', '23'] | ['8', '9'] | ['X ', 'X'] | ['8 ', '8'] | ['7', '10'] |
| 0.84(30/36) | CVCL_0291 | HCT 116 | ['10', '11'] | ['10', '12'] | ['11', '12'] | ['11', '13'] | ['17', '21', '22', '23'] | ['8', '9'] | ['X ', 'X'] | ['8', '9'] | ['7', '10'] |
| 0.84(30/36) | CVCL_0291 | HCT 116 | ['10', '11'] | ['10', '12'] | ['11', '12'] | ['11', '13'] | ['17', '21', '22', '23'] | ['8', '9'] | ['X', 'Y'] | ['8 ', '8'] | ['7', '10'] |
| 0.84(30/36) | CVCL_0291 | HCT 116 | ['10', '11'] | ['10', '12'] | ['11', '12'] | ['11', '13'] | ['17', '21', '22', '23'] | ['8', '9'] | ['X', 'Y'] | ['8', '9'] | ['7', '10'] |
| 0.84(30/36) | CVCL_0291 | HCT 116 | ['10', '11'] | ['10', '12'] | ['11', '12'] | ['11', '13'] | ['17', '22'] | ['8', '9'] | ['X ', 'X'] | ['8 ', '8'] | ['7', '9', '10', '11'] |
| 0.84(30/36) | CVCL_0291 | HCT 116 | ['10', '11'] | ['10', '12'] | ['11', '12'] | ['11', '13'] | ['17', '22'] | ['8', '9'] | ['X ', 'X'] | ['8', '9'] | ['7', '9', '10', '11'] |
| 0.84(30/36) | CVCL_0291 | HCT 116 | ['10', '11'] | ['10', '12'] | ['11', '12'] | ['11', '13'] | ['17', '22'] | ['8', '9'] | ['X', 'Y'] | ['8 ', '8'] | ['7', '9', '10', '11'] |
| 0.84(30/36) | CVCL_0291 | HCT 116 | ['10', '11'] | ['10', '12'] | ['11', '12'] | ['11', '13'] | ['17', '22'] | ['8', '9'] | ['X', 'Y'] | ['8', '9'] | ['7', '9', '10', '11'] |
| 0.83(30/36) | STRJ0016 | ACJ-Cells-No. 41 | ['10', '11'] | ['10', '11'] | ['11', '12'] | ['11', '13'] | ['17', '17'] | ['8', '9'] | ['X', 'X'] | ['8', '8'] | ['7', '9'] |

# LOVO STR

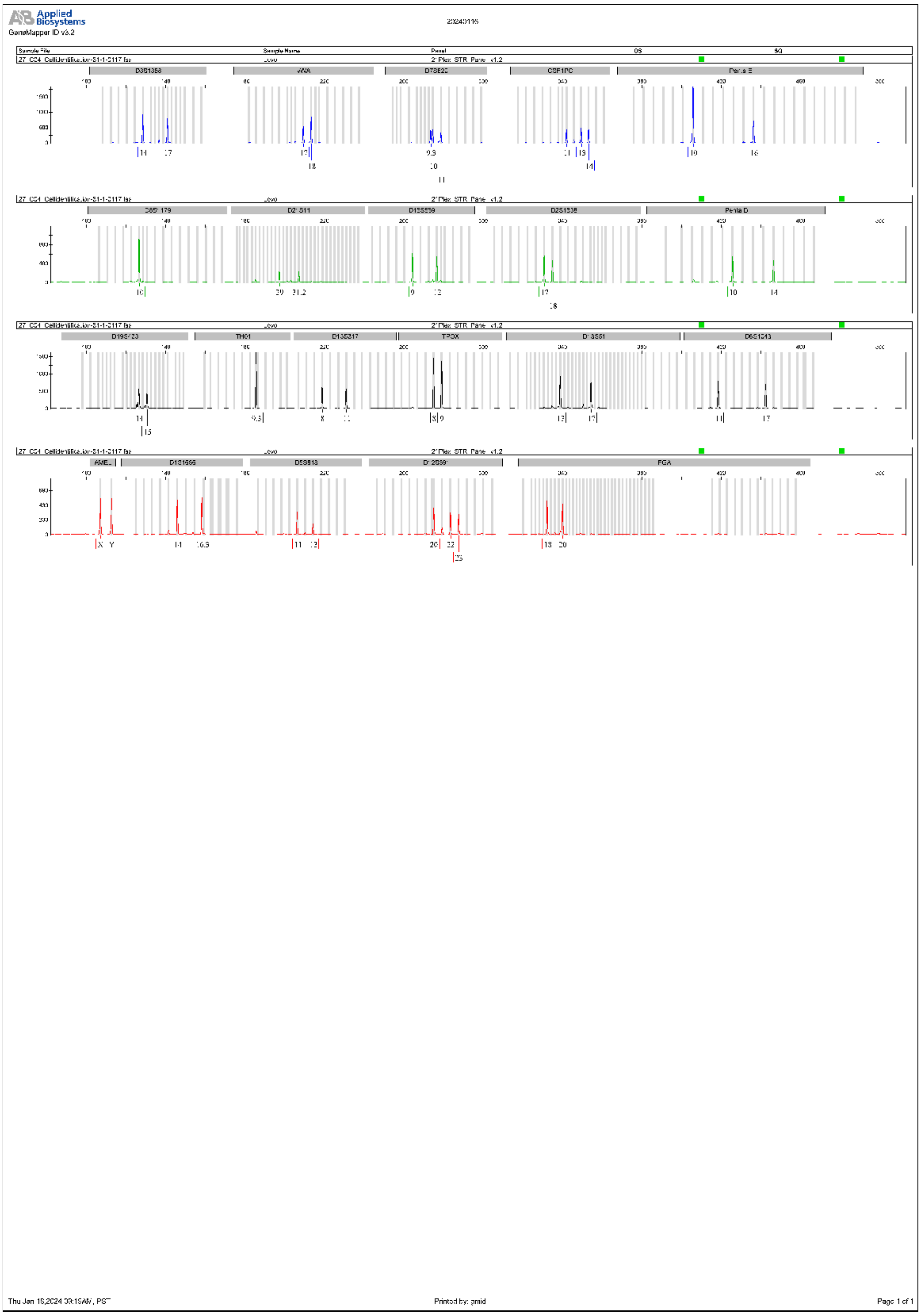
Applied Biosystems
GeneMapper ID v3.2
20240116
Sample File
Sample Name
Panel
OS
SQ
D3S1358
vWA
D7S820
CSF1PO
Penta E
D8S1179
D21S11
D18S51
D2S1338
Penta D
D19S433
TH01
D13S317
TPOX
D16S539
D6S1043
AMEL
D12S391
FGA
Page 1 of 1

| EV | Cell No. | Cell name | Locus names | | | | | | | | |
|---|---|---|---|---|---|---|---|---|---|---|---|
| | | | D5S818 | D13S317 | D7S820 | D16S539 | VWA | TH01 | AM | TPOX | CSF1PO |
| | *Query (Your Cell)* | | 11,13 | 8,11 | 9.3,10,11 | 9,12 | 17,18 | 9.3,9.3 | X,Y | 8,9 | 11,13,14 |
| 1.0(36/36) | CVCL_0399 | LoVo | ['11', '13'] | ['8', '11'] | ['9.3', '10', '11'] | ['9', '12'] | ['17', '18'] | ['9.3', '9.3'] | ['X', 'Y'] | ['8', '9'] | ['11', '13', '14'] |
| 1.0(36/36) | CVCL_4Y03 | LoVo-Luc#2 | ['11', '13'] | ['8', '11'] | ['9.3', '10', '11'] | ['9', '12'] | ['17', '18'] | ['9.3', '9.3'] | ['X', 'Y'] | ['8', '9'] | ['11', '13', '14'] |
| 0.98(35/36) | CVCL_0399 | LoVo | ['11', '12', '13'] | ['8', '11'] | ['9.3', '10', '11'] | ['9', '12'] | ['17', '18'] | ['9.3', '9.3'] | ['X', 'Y'] | ['8', '9'] | ['11', '13', '14'] |
| 0.98(35/36) | CVCL_0399 | LoVo | ['11', '13'] | ['8', '11'] | ['9.3', '10', '11'] | ['9', '11', '12'] | ['17', '18'] | ['9.3', '9.3'] | ['X', 'Y'] | ['8', '9'] | ['11', '13', '14'] |
| 0.98(35/36) | CVCL_0399 | LoVo | ['11', '13'] | ['8', '11'] | ['9.3', '10', '11'] | ['9', '12'] | ['17', '18'] | ['9.3', '9.3'] | ['X', 'Y'] | ['8', '9'] | ['10', '11', '13', '14'] |
| 0.97(35/36) | CVCL_0399 | LoVo | ['11', '13'] | ['8', '11'] | ['9.3', '10', '11'] | ['9', '12'] | ['17', '18'] | ['9.3', '9.3'] | ['X', 'Y'] | ['8', '9'] | ['11', '14'] |
| 0.97(35/36) | CVCL_0399 | LoVo | ['11', '13'] | ['8', '11'] | ['10', '11'] | ['9', '12'] | ['17', '18'] | ['9.3', '9.3'] | ['X', 'Y'] | ['8', '9'] | ['11', '13', '14'] |
| 0.95(34/36) | CVCL_0399 | LoVo | ['11', '12', '13'] | ['8', '11'] | ['9.3', '10', '11'] | ['9', '11', '12'] | ['17', '18'] | ['9.3', '9.3'] | ['X', 'Y'] | ['8', '9'] | ['11', '13', '14'] |
| 0.95(34/36) | CVCL_0399 | LoVo | ['11', '12', '13'] | ['8', '11'] | ['9.3', '10', '11'] | ['9', '12'] | ['17', '18'] | ['9.3', '9.3'] | ['X', 'Y'] | ['8', '9'] | ['10', '11', '13', '14'] |
| 0.95(34/36) | CVCL_0399 | LoVo | ['11', '12', '13'] | ['8', '11'] | ['9.3', '10', '11'] | ['9', '12'] | ['17', '18'] | ['9.3', '9.3'] | ['X', 'Y'] | ['8', '9'] | ['11', '14'] |
| 0.95(34/36) | CVCL_0399 | LoVo | ['11', '12', '13'] | ['8', '11'] | ['10', '11'] | ['9', '12'] | ['17', '18'] | ['9.3', '9.3'] | ['X', 'Y'] | ['8', '9'] | ['11', '13', '14'] |
| 0.95(34/36) | CVCL_0399 | LoVo | ['11', '13'] | ['8', '11'] | ['9.3', '10', '11'] | ['9', '11', '12'] | ['17', '18'] | ['9.3', '9.3'] | ['X', 'Y'] | ['8', '9'] | ['10', '11', '13', '14'] |
| 0.95(34/36) | CVCL_0399 | LoVo | ['11', '13'] | ['8', '11'] | ['9.3', '10', '11'] | ['9', '11', '12'] | ['17', '18'] | ['9.3', '9.3'] | ['X', 'Y'] | ['8', '9'] | ['11', '14'] |
| 0.95(34/36) | CVCL_0399 | LoVo | ['11', '13'] | ['8', '11'] | ['9.3', '10', '11'] | ['9', '12'] | ['16', '17', '18', '19'] | ['9.3', '9.3'] | ['X', 'Y'] | ['8', '9'] | ['11', '13', '14'] |
| 0.95(34/36) | CVCL_0399 | LoVo | ['11', '13'] | ['8', '11'] | ['9.3', '10', '11'] | ['9', '12'] | ['17', '18'] | ['9.3', '9.3'] | ['X', 'Y'] | ['8', '9'] | ['11', '12', '13'] |
| 0.95(34/36) | CVCL_0399 | LoVo | ['11', '13'] | ['8', '11'] | ['9.3', '10', '11'] | ['9', '12'] | ['17', '18'] | ['9.3', '9.3'] | ['X', 'Y'] | ['8', '9'] | ['11', '12', '13', '14', '15'] |
| 0.95(34/36) | CVCL_0399 | LoVo | ['11', '13'] | ['8', '11'] | ['9.3', '10', '11'] | ['9', '12'] | ['17', '19'] | ['9.3', '9.3'] | ['X', 'Y'] | ['8', '9'] | ['11', '13', '14'] |
| 0.95(34/36) | CVCL_0399 | LoVo | ['11', '13'] | ['8', '11'] | ['10', '11'] | ['9', '11', '12'] | ['17', '18'] | ['9.3', '9.3'] | ['X', 'Y'] | ['8', '9'] | ['11', '13', '14'] |
| 0.95(34/36) | CVCL_0399 | LoVo | ['11', '13'] | ['8', '11'] | ['10', '11'] | ['9', '12'] | ['17', '18'] | ['9.3', '9.3'] | ['X', 'Y'] | ['8', '9'] | ['10', '11', '13', '14'] |
| 0.95(34/36) | CVCL_0399 | LoVo | ['11', '13'] | ['8', '11'] | ['10', '11'] | ['9', '12'] | ['17', '18'] | ['9.3', '9.3'] | ['X', 'Y'] | ['8', '9'] | ['11', '14'] |
| 0.93(33/36) | CVCL_0399 | LoVo | ['11', '12', '13'] | ['8', '11'] | ['9.3', '10', '11'] | ['9', '11', '12'] | ['17', '18'] | ['9.3', '9.3'] | ['X', 'Y'] | ['8', '9'] | ['10', '11', '13', '14'] |
| 0.93(33/36) | CVCL_0399 | LoVo | ['11', '12', '13'] | ['8', '11'] | ['9.3', '10', '11'] | ['9', '11', '12'] | ['17', '18'] | ['9.3', '9.3'] | ['X', 'Y'] | ['8', '9'] | ['11', '14'] |
| 0.93(33/36) | CVCL_0399 | LoVo | ['11', '12', '13'] | ['8', '11'] | ['9.3', '10', '11'] | ['9', '12'] | ['16', '17', '18', '19'] | ['9.3', '9.3'] | ['X', 'Y'] | ['8', '9'] | ['11', '13', '14'] |
| 0.93(33/36) | CVCL_0399 | LoVo | ['11', '12', '13'] | ['8', '11'] | ['9.3', '10', '11'] | ['9', '12'] | ['17', '18'] | ['9.3', '9.3'] | ['X', 'Y'] | ['8', '9'] | ['11', '12', '13'] |
| 0.93(33/36) | CVCL_0399 | LoVo | ['11', '12', '13'] | ['8', '11'] | ['9.3', '10', '11'] | ['9', '12'] | ['17', '18'] | ['9.3', '9.3'] | ['X', 'Y'] | ['8', '9'] | ['11', '12', '13', '14', '15'] |
| 0.93(33/36) | CVCL_0399 | LoVo | ['11', '12', '13'] | ['8', '11'] | ['9.3', '10', '11'] | ['9', '12'] | ['17', '19'] | ['9.3', '9.3'] | ['X', 'Y'] | ['8', '9'] | ['11', '13', '14'] |
| 0.93(33/36) | CVCL_0399 | LoVo | ['11', '12', '13'] | ['8', '11'] | ['10', '11'] | ['9', '11', '12'] | ['17', '18'] | ['9.3', '9.3'] | ['X', 'Y'] | ['8', '9'] | ['11', '13', '14'] |
| 0.93(33/36) | CVCL_0399 | LoVo | ['11', '12', '13'] | ['8', '11'] | ['10', '11'] | ['9', '12'] | ['17', '18'] | ['9.3', '9.3'] | ['X', 'Y'] | ['8', '9'] | ['10', '11', '13', '14'] |
| 0.93(33/36) | CVCL_0399 | LoVo | ['11', '13'] | ['8', '11'] | ['9.3', '10', '11'] | ['9', '11', '12'] | ['16', '17', '18', '19'] | ['9.3', '9.3'] | ['X', 'Y'] | ['8', '9'] | ['11', '13', '14'] |
| 0.93(33/36) | CVCL_0399 | LoVo | ['11', '13'] | ['8', '11'] | ['9.3', '10', '11'] | ['9', '11', '12'] | ['17', '18'] | ['9.3', '9.3'] | ['X', 'Y'] | ['8', '9'] | ['11', '12', '13'] |
| 0.93(33/36) | CVCL_0399 | LoVo | ['11', '13'] | ['8', '11'] | ['9.3', '10', '11'] | ['9', '11', '12'] | ['17', '18'] | ['9.3', '9.3'] | ['X', 'Y'] | ['8', '9'] | ['11', '12', '13', '14', '15'] |
| 0.93(33/36) | CVCL_0399 | LoVo | ['11', '13'] | ['8', '11'] | ['9.3', '10', '11'] | ['9', '11', '12'] | ['17', '19'] | ['9.3', '9.3'] | ['X', 'Y'] | ['8', '9'] | ['11', '13', '14'] |
| 0.93(33/36) | CVCL_0399 | LoVo | ['11', '13'] | ['8', '11'] | ['9.3', '10', '11'] | ['9', '12'] | ['16', '17', '18', '19'] | ['9.3', '9.3'] | ['X', 'Y'] | ['8', '9'] | ['10', '11', '13', '14'] |

# SW620 STR

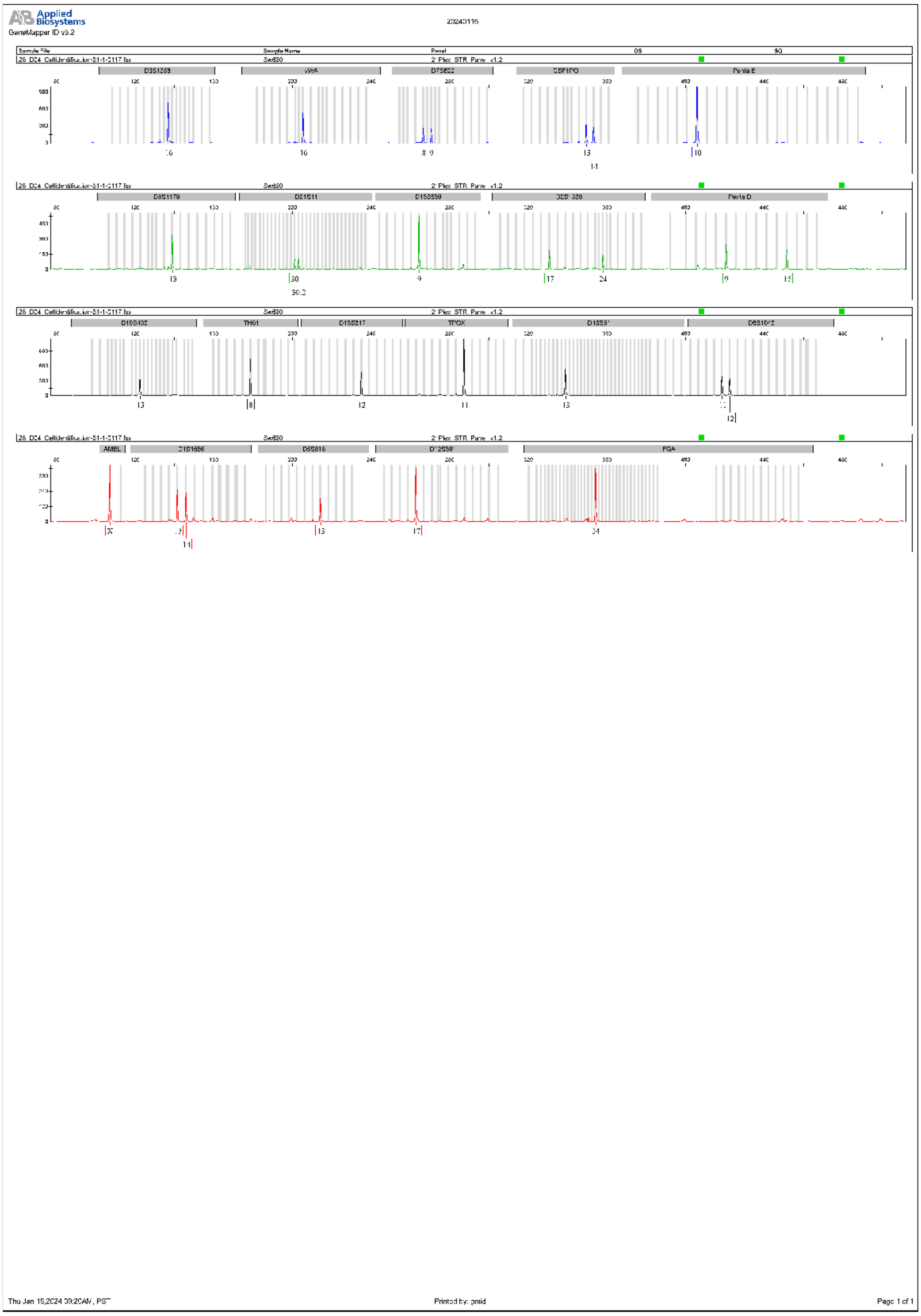
Applied Biosystems
GeneMapper ID v3.2
Sample File
Sample Name
Panel
OS
SQ
vWA
CSF1PO
Penta E
Penta D
TH01
TPOX
AMEL
FGA
Page 1 of 1

| EV | Cell No. | Cell name | Locus names | | | | | | | | |
|---|---|---|---|---|---|---|---|---|---|---|---|
| | | | D5S818 | D13S317 | D7S820 | D16S539 | VWA | TH01 | AM | TPOX | CSF1PO |
| | ***Query (Your Cell)*** | | 13,13 | 12,12 | 8,9 | 9,9 | 16,16 | 8,8 | X,X | 11,11 | 13,14 |
| 0.94(34/36) | CCL-227 | SW620 [SW-620] | ['13', '13'] | ['12', '12'] | ['8', '9'] | ['9', '13'] | ['16', '16'] | ['8', '8'] | ['X', 'X'] | ['11', '11'] | ['13', '14'] |
| 1.0(36/36) | STRJ0025 | ACJ-Cells-No. 78 | ['13', '13'] | ['12', '12'] | ['8', '9'] | ['9', '9'] | ['16', '16'] | ['8', '8'] | ['X', 'X'] | ['11', '11'] | ['13', '14'] |
| 1.0(36/36) | CVCL_J268 | SW620-Luc | ['13', '13'] | ['12', '12'] | ['8', '9'] | ['9', '9'] | ['16', '16'] | ['8', '8'] | ['X', 'X'] | ['11', '11'] | ['13', '14'] |
| 0.94(34/36) | HPACC | SW-620 | ['13', '13'] | ['12', '12'] | ['8', '9'] | ['9', '13'] | ['16', '16'] | ['8', '8'] | ['X', 'X'] | ['11', '11'] | ['13', '14'] |
| 0.94(34/36) | KCLB-Korea-10227 | SW620; SW-620 | ['13', '13'] | ['12', '12'] | ['8', '9'] | ['9', '13'] | ['16', '16'] | ['8', '8'] | ['X', 'X'] | ['11', '11'] | ['13', '14'] |
| 0.94(34/36) | KCLB-Korea-60068 | SW620 | ['13', '13'] | ['12', '12'] | ['8', '9'] | ['9', '13'] | ['16', '16'] | ['8', '8'] | ['X', 'X'] | ['11', '11'] | ['13', '14'] |
| 0.94(34/36) | STRJ0019 | ACJ-Cells-No. 47 | ['13', '13'] | ['12', '12'] | ['8', '9'] | ['9', '13'] | ['16', '16'] | ['8', '8'] | ['X', 'X'] | ['11', '11'] | ['13', '14'] |
| 0.94(34/36) | STRJ0045 | ACJ-Cells-No. 47 | ['13', '13'] | ['12', '12'] | ['8', '9'] | ['9', '13'] | ['16', '16'] | ['8', '8'] | ['X', 'X'] | ['11', '11'] | ['13', '14'] |
| 0.94(34/36) | CVCL_8092 | BIC-1 | ['13', '13'] | ['12', '12'] | ['8', '9'] | ['9', '13'] | ['16', '16'] | ['8', '8'] | ['X', 'X'] | ['11', '11'] | ['13', '14'] |
| 0.94(34/36) | CVCL_0547 | SW620 | ['13', '13'] | ['12', '12'] | ['8', '9'] | ['9', '13'] | ['16', '16'] | ['8', '8'] | ['X', 'X'] | ['11', '11'] | ['13', '14'] |
| 0.89(32/36) | CRL-7940 | SW-527 | ['13', '13'] | ['12', '12'] | ['8', '8'] | ['9', '13'] | ['16', '16'] | ['8', '8'] | ['X', 'X'] | ['11', '11'] | ['13', '14'] |
| 0.89(32/36) | KCLB-Korea-60067 | SW480E | ['13', '13'] | ['12', '12'] | ['8', '8'] | ['9', '13'] | ['16', '16'] | ['8', '8'] | ['X', 'X'] | ['11', '11'] | ['13', '14'] |
| 0.89(32/36) | CVCL_3799 | SW527 | ['13', '13'] | ['12', '12'] | ['8', '8'] | ['9', '13'] | ['16', '16'] | ['8', '8'] | ['X', 'X'] | ['11', '11'] | ['13', '14'] |
| 0.89(32/36) | CVCL_0547 | SW620 | ['13', '13'] | ['12', '12'] | ['8', '9'] | ['13 ', '13'] | ['16', '16'] | ['8', '8'] | ['X', 'X'] | ['11', '11'] | ['13', '14'] |
| 0.86(31/36) | CVCL_AT67 | SW480E | ['13', '13'] | ['12', '12'] | ['8', '8'] | [''] | ['16', '16'] | ['8', '8'] | ['X', 'X'] | ['11', '11'] | ['13', '14'] |
| 0.83(30/36) | ACC-313 | SW-480 | ['13', '13'] | ['12', '12'] | ['8', '8'] | ['13', '13'] | ['16', '16'] | ['8', '8'] | ['X', 'X'] | ['11', '11'] | ['13', '14'] |
| 0.83(30/36) | CCL-228 | SW480 [SW-480] | ['13', '13'] | ['12', '12'] | ['8', '8'] | ['13', '13'] | ['16', '16'] | ['8', '8'] | ['X', 'X'] | ['11', '11'] | ['13', '14'] |
| 0.83(30/36) | CRL-2176 | SW-598 | ['13', '13'] | ['12', '12'] | ['8', '8'] | ['13', '13'] | ['16', '16'] | ['8', '8'] | ['X', 'X'] | ['11', '11'] | ['13', '14'] |
| 0.83(30/36) | HPACC | SW-480 | ['13', '13'] | ['12', '12'] | ['8', '8'] | ['13', '13'] | ['16', '16'] | ['8', '8'] | ['X', 'X'] | ['11', '11'] | ['13', '14'] |
| 0.83(30/36) | KCLB-Korea-10228 | SW480; SW-480 | ['13', '13'] | ['12', '12'] | ['8', '8'] | ['13', '13'] | ['16', '16'] | ['8', '8'] | ['X', 'X'] | ['11', '11'] | ['13', '14'] |
| 0.83(30/36) | CVCL_0546 | SW480 | ['13', '13'] | ['12', '12'] | ['8', '8'] | ['13', '13'] | ['16', '16'] | ['8', '8'] | ['X', 'X'] | ['11', '11'] | ['13', '14'] |
| 0.83(30/36) | CVCL_A9MF | SW480-EGFP | ['13', '13'] | ['12', '12'] | ['8', '8'] | ['13', '13'] | ['16', '16'] | ['8', '8'] | ['X', 'X'] | ['11', '11'] | ['13', '14'] |
| 0.83(30/36) | CVCL_A4BX | SW480-Luc2 | ['13', '13'] | ['12', '12'] | ['8', '8'] | ['13', '13'] | ['16', '16'] | ['8', '8'] | ['X', 'X'] | ['11', '11'] | ['13', '14'] |
| 0.83(30/36) | CVCL_A9MG | SW480-Luc2-tdT | ['13', '13'] | ['12', '12'] | ['8', '8'] | ['13', '13'] | ['16', '16'] | ['8', '8'] | ['X', 'X'] | ['11', '11'] | ['13', '14'] |
| 0.83(30/36) | CVCL_A9MH | SW480-mCherry | ['13', '13'] | ['12', '12'] | ['8', '8'] | ['13', '13'] | ['16', '16'] | ['8', '8'] | ['X', 'X'] | ['11', '11'] | ['13', '14'] |
| 0.83(30/36) | CVCL_A9MI | SW480-tdT | ['13', '13'] | ['12', '12'] | ['8', '8'] | ['13', '13'] | ['16', '16'] | ['8', '8'] | ['X', 'X'] | ['11', '11'] | ['13', '14'] |
| 0.83(30/36) | CVCL_F649 | SW598 | ['13', '13'] | ['12', '12'] | ['8', '8'] | ['13', '13'] | ['16', '16'] | ['8', '8'] | ['X', 'X'] | ['11', '11'] | ['13', '14'] |
| 0.83(30/36) | CVCL_A9MJ | SW620-EGFP | ['13', '13'] | ['12', '12'] | ['8', '8'] | ['13', '13'] | ['16', '16'] | ['8', '8'] | ['X', 'X'] | ['11', '11'] | ['13', '14'] |
| 0.83(30/36) | CVCL_A9MK | SW620-Luc2-tdT | ['13', '13'] | ['12', '12'] | ['8', '8'] | ['13', '13'] | ['16', '16'] | ['8', '8'] | ['X', 'X'] | ['11', '11'] | ['13', '14'] |
| 0.83(30/36) | CVCL_A9ML | SW620-mCherry | ['13', '13'] | ['12', '12'] | ['8', '8'] | ['13', '13'] | ['16', '16'] | ['8', '8'] | ['X', 'X'] | ['11', '11'] | ['13', '14'] |
| 0.83(30/36) | CVCL_A9MM | SW620-tdT | ['13', '13'] | ['12', '12'] | ['8', '8'] | ['13', '13'] | ['16', '16'] | ['8', '8'] | ['X', 'X'] | ['11', '11'] | ['13', '14'] |
| 0.83(30/36) | CVCL_3884 | SW742 | ['13', '13'] | ['12', '12'] | ['8', '9'] | ['12', '12'] | ['16', '16'] | ['8', '8'] | ['X', 'X'] | ['11', '11'] | ['13', '13'] |
| 0.72(26/36) | CRL-2547 | Panc-10.05 | ['13', '13'] | ['12', '12'] | ['8', '9'] | ['9', '12'] | ['16', '16'] | ['6', '9.3'] | ['X', 'X'] | ['11', '11'] | ['12', '12'] |
| 0.72(26/36) | CRL-2558 | PL45 | ['13', '13'] | ['12', '12'] | ['8', '9'] | ['9', '12'] | ['16', '16'] | ['6', '9.3'] | ['X', 'X'] | ['11', '11'] | ['12', '12'] |
| 0.72(26/36) | CVCL_1639 | Panc 10.05 | ['13', '13'] | ['12', '12'] | ['8', '9'] | ['9', '12'] | ['16', '16'] | ['6', '9.3'] | ['X', 'X'] | ['11', '11'] | ['12', '12'] |
| 0.72(26/36) | CVCL_3567 | PL45 | ['13', '13'] | ['12', '12'] | ['8', '9'] | ['9', '12'] | ['16', '16'] | ['6', '9.3'] | ['X', 'X'] | ['11', '11'] | ['12', '12'] |
| 0.67(24/36) | CVCL_VV25 | BT169 | ['13', '13'] | ['12', '12'] | ['9', '9'] | ['9', '9'] | ['16', '16'] | ['6', '9.3'] | ['X', 'X'] | ['9', '11'] | ['11', '12'] |
| 0.67(24/36) | CVCL_9V18 | DFW | ['11', '11'] | ['12', '12'] | ['8', '9'] | ['9', '12'] | ['16', '16'] | ['7', '7'] | ['X', 'X'] | ['11', '11'] | ['13', '13'] |
| 0.61(22/36) | CRL-5837 | NCI-H719 [H719] | ['13', '13'] | ['12', '12'] | ['10', '10'] | ['9', '9'] | ['16', '18'] | ['8', '9'] | ['X', 'X'] | ['8', '11'] | ['10', '11'] |
| 0.61(22/36) | CRL-7578 | Hs-846.Sk | ['13', '13'] | ['12', '12'] | ['8', '9'] | ['12', '12'] | ['16', '16'] | ['7', '9'] | ['X', 'X'] | ['8', '8'] | ['12', '13'] |
